# Using 3D and 4D digital human modeling in extended reality-based rehabilitation: a systematic review

**DOI:** 10.3389/fbioe.2025.1496168

**Published:** 2025-03-12

**Authors:** Mengdi Lu, Wim Saeys, Maria Maryam, Inva Gjeleshi, Hoda Nazarahari, Steven Truijen, Sofia Scataglini

**Affiliations:** ^1^ 4D4ALL Laboratory, Department of Rehabilitation Sciences and Physiotherapy, Center for Health and Technology (CHaT), Faculty of Medicine and Health Sciences, University of Antwerp, Antwerp, Belgium; ^2^ Department of Neurological Rehabilitation, Rehabilitation Hospital Revarte, Edegem, Belgium

**Keywords:** 3D, 4D, digital human modeling, digital twin, extended reality, virtual reality, augmented reality, rehabilitation

## Abstract

**Introduction:**

Extended reality (XR) is increasingly used in rehabilitation, showing potential to enhance clinical outcomes. Recently, integrating digital human modeling (DHM) with XR has gained attention. This systematic review aimed to evaluate the effectiveness of combining 3D and 4D DHM with XR in rehabilitation.

**Methods:**

A systematic literature search was conducted according to PRISMA 2020 guidelines on the 28^th^ of May 2024 in five databases (PubMed, IEEE Database, Cochrane Library, Web of Science, and Science Direct). All types of experimental studies investigating the effectiveness of XR using 3D and 4D DHM in rehabilitation were included. Consensus-based Standards for the selection of health Measurement Instruments (COSMIN) and Evidence-Based Guideline Development (EBRO) were used to evaluate the methodological quality of the studies included.

**Results:**

Of the 1048 articles found, 16 were included in this review. These studies focused on 3D DHM in XR-based rehabilitation across various conditions and demonstrated superior effectiveness, especially in individuals with neglect, anorexia nervosa, bulimia nervosa, and type 2 diabetes in comparison with conventional therapy. DHM, captured via 3D cameras and combined with motion analysis or Wii remotes, was integrated into XR systems like VR games and avatar therapy. The studies reveal positive impacts on functional (e.g., upper limb function, gait, balance, quality of life), physical (e.g., pain reduction, spasticity, joint range), psychological (e.g., depression, emotional regulation, body image), and general health outcomes (e.g., body composition, metabolic health).

**Conclusion:**

Despite variability in study parameters, limited evidence suggests that 3D DHM in XR-based rehabilitation may enhance physical and psychological recovery across various pathologies. This review highlights the potential of DHM and XR integration but underscores the need for further research with larger samples, longer follow-ups, and standardized measures to confirm these technologies’ reliability and effectiveness in rehabilitation.

**Systematic Review Registration:**

https://www.crd.york.ac.uk/PROSPERO/view/CRD42024553551, identifier CRD42024553551.

## 1 Introduction

Digital rehabilitation is a rapidly expanding discipline that involves using digital technology to provide treatments for the rehabilitation process that are affordable, accessible, and user-friendly (Mehl et al., 2018). In contrast to traditional therapy, it seeks to deliver new perspectives on person-centered training and a variety of novel experiences during rehabilitation (Robbins et al., 2018). The field of extended reality (XR) technology is one example of advancements; it creates an interactive experience between the digital and physical worlds (Wonggom et al., 2020).

XR is an umbrella term for immersive technologies, including virtual reality (VR), augmented reality (AR), and mixed reality (MR) (Tu et al., 2023). VR refers to a computer-generated, immersive environment that enables users to interact with digital content and mimics real-world experiences in a virtual environment (Henderson et al., 2007; Weiss et al., 2006; Schultheis and Rizzo, 2001; Wilson et al., 1997). It can be categorized into three levels of immersion: non-immersive experiences, which are delivered through desktop screens; semi-immersive experiences, which involve projection displays; and fully immersive experiences, which rely on head-mounted displays (HMD) to provide a comprehensive sensory environment (Mujber et al., 2004). AR adds digital content to the physical world, enriching real-life experiences by overlaying virtual information on physical objects in real space (Vinolo et al., 2021). In comparison, MR is represented as a more sophisticated iteration of VR and AR by adding interactive experiences with virtual objects in a real-world environment (Palumbo et al., 2022). MR users experience the physical and virtual content co-existing, and the virtual objects as actually present in their physical environment (Žilak et al., 2022). Consequently, XR technology enables the customization of all 3D components in the space, not only objects but also environments, therefore it improves the ability to observe and evaluate actions in real-world settings while in rehabilitation training (Goh et al., 2024). Moreover, XR has been widely proven to offer added value during rehabilitation treatment in patients’ clinical outcomes, such as enhanced motor functions (Laver et al., 2017; Mubin et al., 2019; Saposnik and Levin, 2011), increased community participation (Gorman and Gustafsson, 2022), and improved psychological and cognitive wellbeing (Massetti et al., 2018).

As one of the most used personalized elements in XR training programs, avatars are often added to provide various real-time virtual feedback (Dewez et al., 2021; Liu et al., 2022) and embody human presence in a virtual environment for interactive and effective rehabilitation during training (Wonggom et al., 2020). Numerous studies have highlighted the efficacy of avatars in promoting motor recovery (Liu et al., 2022; Liu et al., 2020) and supporting mental health (Burton et al., 2015). These avatars not only facilitate profound higher levels of user engagement with the virtual environment but also play a crucial role in motor skill reacquisition by enabling self-correction through avatar observation, thereby improving clinical rehabilitation outcomes (Liu et al., 2020; Numa et al., 2015).

Building upon the basic benefits of avatars in XR, this technology has gradually advanced towards 3D and 4D Digital Human Modeling (DHM), which represents human beings with physical appearance, movements, and behaviors in computerized, digital, and virtual visualization (Scataglini and Paul, 2019). While conventional avatars cannot offer the same high realism and lifelike interactions in virtual spaces as 3D and 4D DHM, 3D DHM can only represent a static visualization with fixed parameters, such as body shape (Scataglini and Paul, 2019). DHM in 4D, on the other hand, enables a dynamic capture of the full body shape with both 3D appearance and changes followed by time during a motor task. As a result, the benefits of DHM encourage and facilitate many research projects that aim to investigate this technology’s efficacy in enhancing clinical outcomes and the rehabilitation process.

While conventional avatars cannot offer the same high realism and lifelike interactions in virtual spaces as 3D and 4D DHM, 3D DHM can only represent a static visualization with fixed parameters, such as body shape (Scataglini and Paul, 2019). DHM in 4D, on the other hand, enables a dynamic capture of the full body shape with both 3D appearance and changes followed by time during a motor task. As a result, the benefits of DHM encourage and facilitate many research projects that aim to investigate this technology’s efficacy in enhancing clinical outcomes and the rehabilitation process.

Moreover, Massetti et al. (2018) evaluated the training programs that integrated VR with 3D DHM in people with spinal cord injuries and the results highlighted the potential of using DHM to promote strength, balance, gait, and motor recovery after rehabilitation. A more recent systematic review and meta-analysis by Scataglini et al. (2024) evaluated the accuracy, validity, and reliability of Markerless Camera-Based 3D Motion Capture Systems (MCBS) versus Marker-Based 3D Motion Capture Systems in gait analysis. Spatiotemporal parameters demonstrated excellent accuracy, validity, and reliability, with moderate agreement in hip and knee kinematic variables. While 3D digital health models were produced in both cases, the MCBS, with its 3D and 4D scanning capabilities, proved to be a more comprehensive tool for creating a personalized digital human model that considers size, form, and aesthetics while integrating gait analysis and rehabilitation into inclusive precision medicine.

Another systematic review (Chattopadhyay et al., 2020) also found evidence supporting the efficacy of integrating computer-controlled 3D DHM in patient-facing systems, demonstrating their ability to significantly enhance health outcomes in various types of target populations compared to traditional interventions, such as quality of life (Andrade et al., 2015), physical activity (Bickmore et al., 2013; Ellis et al., 2013), mental health (Wieser et al., 2010), psychological condition (Cassidy et al., 2016), patient education (Gunn et al., 2020). Additionally, only two gait analysis studies were found related to 4D DHM. De Rosario et al. (2023) (De Rosario et al., 2023) presented the biomechanical applications of using 4D scanning technologies, especially in assessing volumetric asymmetries and rehabilitation. While in another study by Meletani et al. (2024) (Meletani et al., 2024) demonstrated the 4D scanning system has comparable reliability and accuracy to inertial measurement unit systems in gait analysis. Both studies confirmed the validity and reliability of 4D DHM and their potential use for measuring biomechanical characteristics in rehabilitation. However, as 4D DHM is a novel method of rehabilitation, no reviews have been found that have thoroughly covered therapy and assessments in XR-based applications.

Therefore, the purpose of this systematic review was to present the first thorough analysis of current training approaches that use 3D–4D DHM in XR–based applications for rehabilitation. Regarding 3D–4D DHM in XR-based rehabilitation, this study made a distinctive contribution to identifying the gaps in up-to-date utilization of the technologies in clinical settings and inspiring further investigations and education by researchers and professionals into 3D-4D DHM in XR-based rehabilitation.

## 2 Methods

### 2.1 Search strategy

A systematic literature review was performed from inception until 28 May 2024, in five electronic databases: PubMed, IEEE Database, Cochrane Library, Web of Science (WOS), and Science Direct, to identify relevant studies. This systematic review was carried out and reported following the Preferred Reporting Items for Systematic Reviews (PRISMA) (Moher et al., 2009) statement and Health Literacy (Meeus and Gebruers, 2021). The protocol of this review was registered in the International Prospective Register of Systematic Reviews, PROSPERO (CRD42024553551), and can be consulted online (www.crd.york.ac.uk/prospero/).

A comprehensive keyword search was conducted, incorporating terms related to XR, 3D-4D DHM, and performance. Additionally, a manual search of review references was conducted to identify relevant studies. A detailed list of search strategies and the number of hits for each database can be found in Supplementary Appendix 1.

### 2.2 Eligibility criteria

The criteria are guided by the PICO framework.

- P: People who followed rehabilitation programs or were admitted to the rehabilitation department.
- I: Any form of combination of XR-based intervention and DHM for rehabilitation purposes was included. Any surgeries, reconstructions, 3D printing, and chatbots without XR application were excluded.
- C: No limitation for the comparisons, depending on the included studies.
- O: Outcome measures focused on performance, balance, posture, gait, and education were included.
- S: The systematic reviews, meta-analyses, case reports, and letters were excluded.
- T: This search has no restriction on the publication date and was restricted to clinical studies published in English and involving human subjects.

### 2.3 Data collection and extraction

Four authors (H.N., I.G.J., M.L., M.M.) independently searched the article titles and abstracts for the initial screening. The screening was processed on Rayyan, a web-based application for conducting systematic review, to reduce any potential bias during screening.

For the second screening, the full text of the articles was then evaluated, and relevant studies were obtained based on the eligibility criteria. Disagreements were resolved by a group discussion until a consensus was made. The following information will be extracted from each included study (Mehl et al., 2018): First author and Publication year (Robbins et al., 2018); Study design (Wonggom et al., 2020); Participant’s characteristics (including types of impairment, number of participants, age, and gender) (Tu et al., 2023); Intervention (including content, frequency and/or duration) (Henderson et al., 2007); Presentation Device and Tracking/Control system (Weiss et al., 2006); Types of reality, stimulation, and avatar as intervention (Schultheis and Rizzo, 2001); Outcome measures (Wilson et al., 1997); Significant results and interpretation.

### 2.4 Assessment of methodological quality

The risk of bias in the included studies was evaluated using the COSMIN (Consensus-based Standards for the Selection of Health Measurement Instruments) guidelines (COSMIN, 2021), which are designed to assess the quality of studies focusing on the reliability and measurement error of outcome measurement instruments (Mokkink et al., 2020). This tool consists of two components: one for reliability and the other for measurement error (Mokkink et al., 2020). The reliability component includes five standards related to design requirements, one addressing “other flaws,” and three standards pertaining to preferred statistical methods. Similarly, the measurement error component includes these same standards, with the addition of two further standards specific to preferred statistical methods for continuous scores and for dichotomous, nominal, or ordinal scores. Each standard is rated on a four-point scale: “very good,” “adequate,” “doubtful,” or “inadequate.” To determine the overall quality of a study in terms of measurement error or reliability, we applied the worst-score-counts method (Mokkink et al., 2010). The quality of the studies included was assessed individually by two of our reviewers (H.N., I.G.J, M.M.) and disagreements, if any, were resolved with another reviewer (M.L.), who facilitated and led the discussion process.

Additionally, all included studies were also graded on methodological quality using the EBRO (Evidence-Based Guideline Development) method (Burgers and van Everdingen, 2004).

## 3 Results

### 3.1 Study selection

After the electronic search of five databases (PubMed, IEEE Database, Cochrane Library, WOS, and Science Direct) and removing the duplicates, 965 articles remained. After reviewing titles and abstracts, seventy articles were retained for full-text review. A total of 54 relevant studies were initially drafted and further excluded after group deliberation. Finally, sixteen articles were included in this review.

**FIGURE 1:**
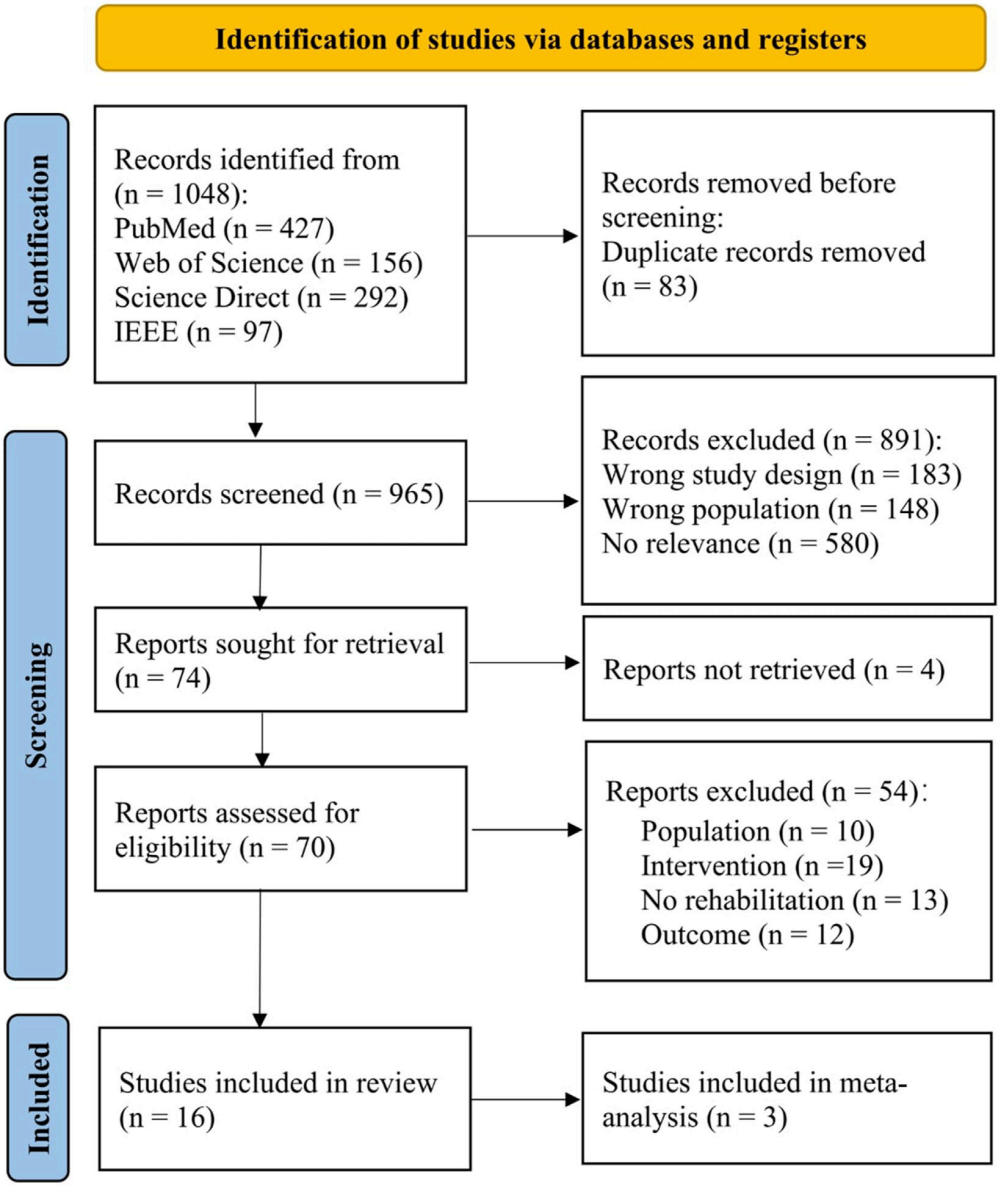
PRISMA flow diagram.

A detailed literature search and study selection process are presented in Figure 1. The flowchart was created using the PRISMA 2020 flow diagram for new systematic reviews which included searches of databases and registers only (Page et al., 2021).

### 3.2 Risk of bias (RoB)

The quality of the included studies was evaluated individually by three reviewers (H.N., I.G.J., M.M.), and disagreements, if any, were resolved through discussion with another reviewer (M.L.), who facilitated and led the discussion process. The RoB for each article is presented in Table 1.

**TABLE 1:** Summary of RoB Assessment based on COSMIN tool and EBRO level, (IA, inadequate, D, doubtful, A, adequate, VG, very good).

Study (year)	Reliability	Measurement errors	Scoring	Overall RoB	EBRO level
Perpiñá et al. (2009)	D	A	A	Moderate	B
Kim et al. (2007)	A	D	D	Moderate	B
Hall et al. (2011)	D	IA	IA	High	C
Tsekleves et al. (2016)	D	D	D	Moderate	C
Keizer et al. (2016)	VG	VG	VG	Low	B
Nosek et al. (2016)	D	IA	IA	High	C
Falconer et al. (2017)	IA	VG	IA	High	C
Nuic et al. (2018)	D	D	D	Moderate	C
Alemanno et al. (2019)	IA	D	IA	High	C
Booth et al. (2019)	D	D	D	Moderate	B
Kammler-Sücker et al. (2021)	D	D	D	Moderate	C
Barhorst-Cates et al. (2022)	D	D	D	Moderate	B
Zhu et al. (2022)	D	D	D	Moderate	C
Mitchell et al. (2023)	D	D	D	Moderate	B
Sansoni et al. (2024)	IA	D	IA	High	A2
Xu et al. (2024)	D	D	D	Moderate	C

The quality was evaluated by the COSMIN tool based on reliability, measurement errors, and scoring. We utilized a system of rating studies where very good scores were considered low risk, adequate scores were viewed as having some concerns, and inadequate or doubtful scores were high risk. Measurement of reliability was classified as 6.3% low RoB, 6.3% with some concerns, and 87.5% high RoB, while measurement errors of the reported results were evaluated as 12.5% low risk of bias, 6.3% with some concerns, and 81.3% high RoB. The scoring of the methodological quality ranged from inadequate to very good. Finally, based on the worst-score-counts method, ten studies were scored as doubtful (Mitchell et al., 2023; Xu et al., 2024; Perpiñá et al., 2009; Tsekleves et al., 2016; Kim et al., 2007; Booth et al., 2019; Barhorst-Cates et al., 2022; Zhu et al., 2022; Nuic et al., 2018; Kammler-Sücker et al., 2021), five studies were inadequate (Nosek et al., 2016; Alemanno et al., 2019; Hall et al., 2011; Falconer et al., 2017; Sansoni et al., 2024), and one study was very good (Keizer et al., 2016). Therefore, the overall RoB varied from moderate to high, except for Keizer et al. (2016) (Keizer et al., 2016) was graded as low RoB.

In addition to the COSMIN tool, each study was further evaluated according to EBRO criteria (Burgers and van Everdingen, 2004). Six studies (Mitchell et al., 2023; Perpiñá et al., 2009; Kim et al., 2007; Booth et al., 2019; Barhorst-Cates et al., 2022; Keizer et al., 2016) were graded as a B level, and nine (Xu et al., 2024; Tsekleves et al., 2016; Zhu et al., 2022; Nuic et al., 2018; Kammler-Sücker et al., 2021; Nosek et al., 2016; Alemanno et al., 2019; Hall et al., 2011; Falconer et al., 2017) had a level of C. Only one study was graded as level A2 (Sansoni et al., 2024).

### 3.3 Study characteristics

Characteristics of the included studies and descriptive data are presented in Table 2, including study design, demographic information, intervention content, and intervention frequency and/or duration from all included studies.

**TABLE 2:** Table of study characteristics.

References	Study Design	Participants impairment	Number of participants (N) (Male/Female)	Mean Age (Years) (SD)/Age range (Years)	Intervention	Intervention Frequency/Duration
Perpiñá et al. (2009)	Case-control study	People with AN and BN	N = 13 EG: N = 8 (4 AN; 4 BN) CG: N = 5 (3 AN; 2 BN)	EG: 18.38 (2.9) CG: 16.6 (1.3)	EG: Virtual environment + training program + standard BI session CG: Relaxation session + standard BI session	Frequency: E.G.,: 1x per week CG: 1x per week Duration: EG: 1h/1x, 6 weeks CG: 3h/1x, 8 weeks
Kim et al. (2007)	Case-control study	People with unilateral neglect caused by stroke	N = 50 E.G.,: N = 10 right hemispheric stroke + left visual neglect CG1: N = 10 HC computer-unfriendly CG2: N = 20 HC computer-friendly	EG: 51.4 (16.3) CG1: 59.8 (5.0) CG2: 29.7 (2.3)	EG and CG1 and CG2: Virtual street environment + crossing street training	Duration: 1 training session

Hall et al. (2011)	Single-group longitudinal study (exploratory study)	People with mild to severe intellectual disabilities	N = 20 (11/9)	20–80	Virtual hospital environment + healthcare tour	Duration: Unlimited, 1 tour session
Tsekleves et al. (2016)	Single case study	People with stroke	N = 1	31	Nintendo Wii + stroke intervention	Frequency: 3x per week Duration: 50 min/1x, 2 weeks

Keizer et al. (2016)	Case-control study	People with AN	N = 59 (0/59) E.G.,: 30 AN CG: 29 HC	EG: 22.03 (3.67) CG: 21.07 (2.34)	EG and CG: Virtual environment + FBI + synchronous/asynchronous perception conditions	Duration: 1 session

Nosek et al. (2016)	Pre-post experimental study	People with a physical disability or chronic health condition	N = 19 (0/19)	22–71	SESL: Virtual environment + avatar + group training	Duration: 7 sessions
Falconer et al. (2017)	Pre-post experimental study	People with BPD	N = 15 (3/12) Final sample N = 11	20–43	E.G.,: Virtual environment + avatar-MBT: MBT + adjunctive avatar therapy CG: MBT without avatar therapy	E.G.,&CG: 10 weeks of introductory sessions. Frequency: 1x per week Duration: 40–60 min/1x, 4x

Nuic et al. (2018)	Pre-post experimental study	People with PD	N = 10 (5/5)	64.2 (6.1)	Toap run video game (Anti PD treatment) 3 motor rehabilitation games +3 different scenarios	Frequency: 3x per week Duration: 6 weeks
Alemanno et al. (2019)	Pre-post experimental study	People with chronic low back pain	N = 20 (9/11)	47.5 (15.3)	VRRS: Virtual environment + sensorimotor rehabilitation trunk exercises	Frequency: 2–3x per week Duration: 1h/1x, 12x
Booth et al. (2019)	Case-control study	Children with CP	N = 66 E.G.,: N = 25 (16/9) CP CG: N = 41 (24/17) HC	EG: 10.4 (2.9) CG: 10 (3)	EG, and CG: Walk on a dual-belt instrumented treadmill + VR screen + HBM/Avatar-based 8 MM biofeedback system	Duration: 1 embedded training session

Kammler-Sücker et al. (2021)	Pre-post experimental study	People with chronic back pain	N = 33 (6/27) Final sample N = 30	22.3 (3.2)	CAVE + HMD + passthrough vision + imitate movements +4 avatars Avatar 1: abstract stick person Avatar 2: cartoon character Avatar 3: realistic character Avatar 4: personalized avatar designed from 3D scans	Frequency: 5x each movement Duration: 4 movements
Barhorst-Cates et al. (2022)	Case-control study	People with left hemisphere stroke	N = 30 EG: N = 18 (8/10) Stroke CG: N = 12 (4/8) HC	EG: 61.8 (11.4) CG: 62.7 (11.2)	EG and CG: Movement imitation task + Virtual environment + avatar + three point-of-view conditions (first-person view, mirroring, anatomical)	Duration: 1 session, 12 movement trials
Zhu et al. (2022)	Case study	People with HD	N = 20 EG: N = 10 (6/4) HD CG: N = 10 (5/5) HC	EG: 54.3 (12.2) CG: 71.83 (10.55)	EG and CG: Ground walking toward the FLIR infrared camera	Duration: at least 1 valid entire gait cycle

Mitchell et al. (2023)	Cohort study	People with type 2 diabetes	N=300 (0/300) EG: N=146 CG: N=154	EG: 55.20 (9.6) CG: 55.54 (11.5)	EG: Diabetes medical group visits + Virtual environment + avatar CG: Diabetes medical visits + at clinics	Frequency: EG 1&EG 2: 1x per week, 120min/x Duration: EG 1&EG 2: 8 weeks
Sansoni et al. (2024)	RCT	People with BN	N=24 (0/24) EG: N=12 BN CG: N=12 BN	EG: 29.25 (7.75) CG: 28.83 (6.48)	EG: VR-CBT + CBT group session CG: CBT group session	Frequency: EG: CBT groups session: 1x per week VR-CBT: 5x per week CG: 1x per week Duration: EG: CBT groups session: 5 weeks VR-CBT: 1h/1x, 5 weeks CG: 5 weeks

Xu et al. (2024)	Pre-post experimental study	People with dyskinesia	N=13 (6/7)	60.9 (11.3)	E-MI+C-MI E-MI: Virtual environment + FBI + physical and virtual bar stimulation + synchronous visuotactile stimulation C-MI: Virtual environment + FBI + physical and virtual bar stimulation + non-synchronous visuotactile stimulation	Frequency: 10 seconds/x Duration: 30x-35x

8MM, Eight maker model; AN, Anorexia nervosa; BI, Body image; BN, Bulimia nervosa; BPD, Borderline personality disorder; CBT, Cognitive behavioral therapy; CAVE, Cave automatic virtual environment; CG, Control group; C-MI, Control motor imagery task; CP, Cerebral palsy; EG, Experimental group; E-MI, Enhanced motor imagery task; FBI, Full-body illusion; HBM, Human body model; HC, Healthy control(s); HD: Hemiplegia's disease; HMD, Head-mounted display; MBT, Mentalization-based group treatment; PD, Parkinson’s disease; RCT, Randomized controlled trial; SESL, Self-Esteem in Second Life; VR, Virtual reality; VRRS, Virtual reality rehabilitation system; x, Time(s).

Of the sixteen included studies, six were pre-post-experimental studies (Xu et al., 2024; Nuic et al., 2018; Kammler-Sücker et al., 2021; Nosek et al., 2016; Alemanno et al., 2019; Falconer et al., 2017), five were case-control studies (Perpiñá et al., 2009; Kim et al., 2007; Booth et al., 2019; Barhorst-Cates et al., 2022; Keizer et al., 2016), two were case studies (Tsekleves et al., 2016; Zhu et al., 2022), one was a longitudinal study (Hall et al., 2011), one was a cohort (Mitchell et al., 2023) and one was a randomized control trial (RCT) (Sansoni et al., 2024). A total of 674 participants were enrolled across studies, with the number per study ranging from one to three hundred (Mitchell et al., 2023; Tsekleves et al., 2016). (Mitchell et al., 2023; Tsekleves et al., 2016) Five studies (Kim et al., 2007; Booth et al., 2019; Barhorst-Cates et al., 2022; Zhu et al., 2022; Keizer et al., 2016) included healthy controls as a comparison group alongside the patient group, three studies (Mitchell et al., 2023; Perpiñá et al., 2009; Sansoni et al., 2024) had two comparable groups of patients, and the rest studies had no comparison group (Xu et al., 2024; Tsekleves et al., 2016; Nuic et al., 2018; Kammler-Sücker et al., 2021; Nosek et al., 2016; Alemanno et al., 2019; Hall et al., 2011; Falconer et al., 2017). The studies covered almost the entire age range of patients from the smallest ten-year-old children up to eighty-year-old elderly people (Booth et al., 2019; Hall et al., 2011).

Numerous pathologies were investigated in the included studies, we generally classified them into three groups based on participants’ function impairments: Firstly, neurological and neuromuscular conditions, which included neglect (Kim et al., 2007), Parkinson’s disease (PD) (Nuic et al., 2018), stroke (Tsekleves et al., 2016; Keizer et al., 2016), (chronic) low back pain (LBP) (Kammler-Sücker et al., 2021; Alemanno et al., 2019), hemispheric diseases (Zhu et al., 2022), cerebral palsy (CP) (Booth et al., 2019), and dyskinesia (Xu et al., 2024); secondly, psychological and mental health conditions, including borderline personality disorder (BPD) (Falconer et al., 2017), anorexia nervosa (AN) (Perpiñá et al., 2009; Barhorst-Cates et al., 2022), bulimia nervosa (BN) (Perpiñá et al., 2009; Sansoni et al., 2024), intellectual disabilities (Hall et al., 2011); lastly, internal and other diseases, such as type 2 diabetes (Mitchell et al., 2023) and chronic health conditions (Nosek et al., 2016).

Regarding the intervention content, all studies included at least three components in their intervention. Firstly, all studies utilized a virtual environment, such as virtual streets (Kim et al., 2007; Booth et al., 2019; Falconer et al., 2017), virtual rooms (Mitchell et al., 2023; Kammler-Sücker et al., 2021; Alemanno et al., 2019; Falconer et al., 2017; Sansoni et al., 2024; Keizer et al., 2016), virtual hospitals (Hall et al., 2011), and non-specified environments (Xu et al., 2024; Perpiñá et al., 2009; Tsekleves et al., 2016; Barhorst-Cates et al., 2022; Zhu et al., 2022; Nuic et al., 2018; Nosek et al., 2016). Secondly, all participants used a humanlike avatar or DHM as an intervention. Only Kammler-Sücker et al. (2021) involved four different types of avatars: abstract stick person, cartoon character, realistic character, and personalized DHM. The third component was the training program related to target functional impairments, which varied significantly among different studies, including body image sessions (Perpiñá et al., 2009), neglect training (crossing street) (Kim et al., 2007), stroke training (Tsekleves et al., 2016; Nuic et al., 2018), physical exercises (Barhorst-Cates et al., 2022; Kammler-Sücker et al., 2021; Alemanno et al., 2019), gait training and walking (Booth et al., 2019; Zhu et al., 2022), perception test in synchronous and asynchronous conditions (Xu et al., 2024; Keizer et al., 2016), healthcare guidance and training (Mitchell et al., 2023; Nosek et al., 2016; Hall et al., 2011), mentalization-based group treatment (Falconer et al., 2017) and cognitive behavioral therapy (Sansoni et al., 2024).

Seven studies (Mitchell et al., 2023; Perpiñá et al., 2009; Tsekleves et al., 2016; Nuic et al., 2018; Alemanno et al., 2019; Falconer et al., 2017; Sansoni et al., 2024) described the frequency of the intervention, with most participants receiving it one to five times per week. In contrast, the remaining nine studies (Xu et al., 2024; Kim et al., 2007; Booth et al., 2019; Barhorst-Cates et al., 2022; Zhu et al., 2022; Kammler-Sücker et al., 2021; Nosek et al., 2016; Hall et al., 2011; Keizer et al., 2016) did not specify the intervention frequency. Two of these studies reported different measures 10 s per session (Xu et al., 2024) and five repetitions per movement (Kammler-Sücker et al., 2021) which can be considered as lacking frequency data. Six studies reported the duration of one intervention session ranged from 40 min to 120 min (Mitchell et al., 2023; Perpiñá et al., 2009; Tsekleves et al., 2016; Alemanno et al., 2019; Falconer et al., 2017; Sansoni et al., 2024), the rest of the studies were not specified in the paper (Xu et al., 2024; Kim et al., 2007; Booth et al., 2019; Barhorst-Cates et al., 2022; Zhu et al., 2022; Nuic et al., 2018; Kammler-Sücker et al., 2021; Nosek et al., 2016; Hall et al., 2011; Keizer et al., 2016). The overall duration of the rehabilitation program was mentioned in all included studies; however, eight studies were completed in a single training session (Xu et al., 2024; Kim et al., 2007; Booth et al., 2019; Barhorst-Cates et al., 2022; Zhu et al., 2022; Kammler-Sücker et al., 2021; Hall et al., 2011; Keizer et al., 2016), and the remaining studies were conducted over a period of 2–10 weeks (Mitchell et al., 2023; Perpiñá et al., 2009; Tsekleves et al., 2016; Nuic et al., 2018; Nosek et al., 2016; Alemanno et al., 2019; Falconer et al., 2017; Sansoni et al., 2024).

### 3.4 Intervention characteristics

Table 3 presents the intervention details, including the device used, type of reality, avatars, and stimulations.

**TABLE 3:** Table of intervention details.

References	DHM: Presentation Device	DHM: Tracking system/Operating mode	Type of reality	Type of stimulation	Avatar (Designed/Scanned)
Perpina et al. (1999)	Computer	Computer (2D mouse)	Non-immersive VR	Visual	Designed avatar (virtual character)
Kim et al. (2007)	HMD	3 DOF Camera	Fully-immersive VR	Visual and auditory	Designed avatar (2D picture)
Hall et al. (2011)	Computer	Camcorder	Non-immersive VR	Visual and verbal	Designed avatar (virtual character)
Tsekleves et al. (2016)	Wiiimote plus video display	Vicon Mocap system with 11 infrared cameras	non-immersive VR	Visual	Designed avatar (virtual character)
Keizer et al. (2016)	HMD: Oculus Rift DK2	\	fully-immersive VR	Visual	Designed avatar (personalized)
Nosek et al. (2016)	Computer	Computer (keyboard/mouse)	Non-immersive VR	Visual and auditory	Designed avatar (personalized)
Falconer et al. (2017)	Computer	Computer (keyboard/mouse)	Non-immersive VR	Visual	Designed avatar (virtual character)
Nuic et al. (2018)	Computer	Kinetic motion sensor	Non-immersive VR	Visual and auditory	Designed avatar (virtual character)
Alemanno et al. (2019)	High-resolution LCD on a large screen	6 DOF motion-tracking system (Polhemus G4, Vermont, US)	Semi-immersive VR	Visual and acoustic	Designed avatar (virtual character)
Booth et al. (2019)	Immersive VR environment (GRAIL)	10-camera 3D motion capture (Vicon Motion Systems Ltd)	Semi-immersive VR	Visual and auditory	Designed avatar (virtual character)
Kammler-Sucker et al. (2021)	A four-sided CAVE A pair of active shutter glasses	Four-camera optical infrared system Kinetic sensor	Fully-immersive VR	Kinesthetic (physical movement tasks)	Scanned avatar
Barhorst-Cates et al. (2022)	VR headset (HTC Vive)	magnetic motion tracking system (trakSTAR) controllers	Fully-immersive VR	Visual	Designed avatar (virtual character)
Zhu et al. (2022)	Computer	12-camera motion analysis system 3D Motion Capture System	Non-immersive VR	Visual	Designed avatar (virtual character)
Sansoni et al. (2024)	HMD	HMD	Augmented reality	Visual	Designed avatar (virtual character)
Xu et al. (2024)	HMD	\	Fully-immersive VR	Visual and tactile	Designed avatar (virtual character)
Mitchell et al. (2023)	Computer	Computer via camera	Non-immersive VR	Visual and auditory	Designed avatar (virtual character)

CAVE, cave automatic virtual environment; DOF, degrees of freedom; HMD, Head-mounted display; VR, virtual reality.

Our review identified four types of reality integrated with DHM: non-immersive VR, semi-immersive VR, fully immersive VR, and AR. Among these, non-immersive VR was integrated with either a computer screen or a TV screen. Only one study (Tsekleves et al., 2016) presented the DHM with the Nintendo Wii system on a TV screen and the DHM was controlled by the Vicon motion capture system. Seven studies (Perpiñá et al., 2009; Zhu et al., 2022; Nuic et al., 2018; Nosek et al., 2016; Hall et al., 2011; Falconer et al., 2017; Sansoni et al., 2024) presented the DHM on a computer screen, though different tracking methods were used. In three studies (Perpiñá et al., 2009; Nosek et al., 2016; Falconer et al., 2017), participants controlled the DHM via a mouse or keyboard, while in another three studies (Zhu et al., 2022; Hall et al., 2011; Sansoni et al., 2024), high-definition motion capture cameras tracked participants’ movements for DHM control. In the study by Nuic et al. (Nuic et al., 2018), the DHM was controlled using a Kinect motion sensor attached to the participant’s body.

Two studies (Booth et al., 2019; Alemanno et al., 2019) were found to combine a semi-immersive VR environment with DHM, which was displayed through a large screen. Both DHMs in these studies were controlled by a motion capture system, Polhemus G4, and Vicon system respectively (Booth et al., 2019; Alemanno et al., 2019).

While another five studies (Xu et al., 2024; Kim et al., 2007; Barhorst-Cates et al., 2022; Kammler-Sücker et al., 2021; Keizer et al., 2016) were conducted within a fully immersive VR environment. Among the four studies (Xu et al., 2024; Kim et al., 2007; Kammler-Sücker et al., 2021; Keizer et al., 2016) that used HMDs to present the DHM, only Kammler-Sucker et al. (Kammler-Sücker et al., 2021) employed special active shutter glasses to convert a 2D image into a stereoscopic image of participant DHM. In the studies by Xu et al. (Xu et al., 2024) and Keizer et al. (Keizer et al., 2016), participant movements were not tracked, and the interaction with the system was limited to observation. In three studies (Kim et al., 2007; Barhorst-Cates et al., 2022; Kammler-Sücker et al., 2021), motion capture systems were used to track participants’ movements.

AR environment was only used in one study conducted by Sansoni et al. (Sansoni et al., 2024), where an HMD was used both to display the DHM and to track movements for controlling the DHM.

In terms of the type of stimulation, visual stimulation was emphasized in all the included studies. Additionally, auditory (Kim et al., 2007; Booth et al., 2019; Nuic et al., 2018; Nosek et al., 2016; Sansoni et al., 2024), verbal (Hall et al., 2011), tactile (Xu et al., 2024), and kinesthetic (Kammler-Sücker et al., 2021) stimulation were incorporated in several studies alongside visual stimulation to enhance clinical outcomes.

As for avatars/DHM, we classified them into two different types: designed avatars (virtual character and personalized character) and scanned avatars. Designed avatars refer to avatars with a cartoon-like or non-realistic appearance, where a virtual character represents a figure that does not resemble the participants. A personalized character refers to a humanoid avatar with a realistic appearance, which is designed by software. Scanned avatars, on the other hand, also represent humanlike avatars, but with a higher level of realism, the appearance of the avatars is modeled using the participants’ scanned appearance and outfit. No 4D DHM was found in the included articles, the study by Kammler-Sücker et al. (2021) was the only one that included a scanned avatar, which is similar to the 4D DHM, to explore the effectiveness of using avatars with varying degrees of realism and similarity in imitative motor behavior. However, 4D DHM was not suitable for this study, as the personalized DHM with a scanned outfit was not controlled by the participants’ movements but rather followed pre-programmed movements.

### 3.5 Outcome measures

All outcome measurements and significant results are presented in Supplementary Table S1. We generally classified them into six categories: functional, physical, psychological, system-embedded, virtual experience-related, and others.

#### 3.5.1 Functional outcome measures

A total of six studies (Mitchell et al., 2023; Tsekleves et al., 2016; Booth et al., 2019; Zhu et al., 2022; Nuic et al., 2018; Alemanno et al., 2019) evaluated functional outcome measures, including the Fugl-Meyer assessment (FMA) upper limb section, nine-hole peg test (NHPT), motor activity log(MAL) subscale: amount of use (AOU), unified Parkinson’s Disease rating scale (UPDRS) parts II and III; Parkinson’s Disease questionnaire (PDQ-39); axial score; freezing of gait questionnaire; activities and balance confidence scale; gait and balance scale; and various gait parameters (double stance durations, step length, step width, cadence, toe-out angle, stance stage, stride, gait velocity, braking index, anticipatory postural adjustments (APAs), and APA displacement). Additional measures included the Roland and Morris disability questionnaire, the 36-item short-form health survey (SF-36), and physical activity.

Significant improvements were observed in all measures after the intervention, except UPDRS, mediolateral APAs displacement (p > 0.05), step width (p > 0.05), and braking index (p > 0.05), which did not show improvements (Nuic et al., 2018). No significant differences in physical activity were observed when comparing the virtual medical visit to the in-person group visit among people with type II diabetes (3.1, 97.5% CI -6.9 to ∞, p < 0.001) (Mitchell et al., 2023).

Furthermore, in terms of gait parameters, there were two studies (Booth et al., 2019; Zhu et al., 2022) validate the accuracy and usability of the DHM-based system in measuring spatiotemporal gait parameters during gait training. Booth et al. (2019) compared an eight marker DHM model with a common-used human body model in children with CP and children with typically developing, the results showed no significant differences in step length (p = 0.74, p = 0.8) or cadence (p > 0.05, p = 0.4), highlighting the comparable usability of DHM in detecting gait abnormalities in both CP patient and normal controls (Booth et al., 2019). However, a statistically significant but negligible reduction in step width was observed in both groups (p < 0.001, p < 0.001) (Booth et al., 2019). Similarly, Zhu et al. (2022) demonstrated comparable results using a marker-based three-dimensional motion analysis system and a dual-channel cascade pose estimation network. Both systems showed high validity and comparable capabilities in assessing gait spatiotemporal parameters, including stride (r = 0.92), step length (r = 0.94), step width (r = 0.98), toe-out angle (r = 0.95), gait speed (r = 0.97) and stance stage duration (r = 0.95) (Zhu et al., 2022). Significant differences were identified across all gait parameters between healthy older adults and participants with hemiplegia (p < 0.05). Healthy older participants displayed superior gait features in stride (p = 0.003), step length (p = 0.01), step width (p < 0.001), toe-out angle (p < 0.001), gait speed (p = 0.006), and stance phase duration (p = 0.004) compared to participants with hemiplegia (Zhu et al., 2022). These findings suggested that DHM-based system is reliable tool for assessing gait performance and detecting gait abnormalities, therefore, it holds significant potential to become an effective tool in gait training in rehabilitation settings.

#### 3.5.2 Physical outcome measures

There were six studies (Mitchell et al., 2023; Tsekleves et al., 2016; Booth et al., 2019; Kammler-Sücker et al., 2021; Alemanno et al., 2019; Sansoni et al., 2024) investigated the physical outcome measures, including the Modified Ashworth Scale (MAS) for shoulder, elbow, finger, and wrist; pain assessments using the numeric rating scale (NRS), McGill Pain Questionnaire, Brief Pain Inventory, number of words chosen, pain score at worst, and average pain score; global impression of change; the joint range of motion (ROM) for trunk maximal and average rotation, pelvic tilt, obliquity, and rotation, hip flexion, knee flexion, and spinal lateral flexion, extension, and horizontal rotation; repetition index; body mass index (BMI); and hemoglobin A1c (HbA1c).

All measurements indicated positive outcomes with the use of DHM (p < 0.05) with enhancing various physical outcome measures: decreased muscle spasticity (Tsekleves et al., 2016), decreased pain levels (Alemanno et al., 2019), and decreased BMI, with the effects lasting up to 12 months (73). While HbA1c levels also showed a decrease, Mitchell et al. (2024) reported no significant differences between the virtual medical visit with DHM and the in-person group visit in terms of HbA1c reduction (0.2, 97.5% CI -∞ to 0.3, P < 0.001).

However, nonsignificant effectiveness was also found in the included studies. The study from Tsekleves et al. (2016) demonstrated an unchanged score of the spasticity at shoulder and elbow joints and a non-significant reduction in spasticity of finger and wrist joints following the intervention of DHM, with a decrease from three to one (p > 0.05).

Regarding ROM, Booth et al. (2019) reported comparable joint ROM results with minimal differences between 8 MM and HBM systems, suggesting the feasibility of using the 8 MM system with DHM for gait training. The most notable significant difference was a 10-degree reduction in knee flexion (p < 0.001), while other ROM changes, including pelvic tilt, obliquity, rotation, and hip flexion, were less than 5°, which is considered within the acceptable range (McGinley et al., 2009). Additionally, Kammler-Sücker et al. (2021) found that due to the ownership of embodiment serving as a mediator factor, the types of avatars did not significantly affect the spinal ROM in lateral flexion. Additionally, the amount of training showed a small negative significant effect in spinal extension (effect size βz = −0.0947; pPB = 0.014) and a small positive significant effect in spinal horizontal rotation (effect size βz = 0.0817; pSM = 0.0064, pPB = 0.0111) (Kammler-Sücker et al., 2021).

#### 3.5.3 Psychological outcome measures

Psychological outcome measures were reported in seven studies (Perpiñá et al., 2009; Nuic et al., 2018; Nosek et al., 2016; Alemanno et al., 2019; Falconer et al., 2017; Sansoni et al., 2024; Keizer et al., 2016), including assessments such as the Beck Depression Inventory (BDI); Positive and Negative Affect Schedule (PNAS); Body Areas Satisfaction Scale (BASS), Situational inventory of body image dysphoria; Body Image Avoidance Questionnaire (BIAQ); Body image automatic thoughts questionnaire (BIATQ); Fear of putting on weight; Body width estimation in height, hip, shoulder, abdomen, Body circumference in hip, shoulder, abdomen; Center for epidemiologic studies depression scale-10; Rosenberg self-esteem scale (RSES), Hudson index of self-esteem; Generalized self-efficacy scale (GSES); 21-item depression, anxiety and stress scales (DASS); Mentalization questionnaire (MQ); Piper fatigue revised scale (PFRS), Negative and positive emotionality questionnaire (EPN-31), Neuropsychological evaluations: cognition; Diabetes Distress Scale-17 (DDS-17); Subscales of Eating Disorder Inventory (EDI): bulimia (BU), drive for thinness (DT).

Significant improvements were observed in depression (Perpiñá et al., 2009; Nosek et al., 2016; Alemanno et al., 2019), self-estimation (Perpiñá et al., 2009; Nosek et al., 2016), and cognitive function (Bickmore et al., 2013) following the intervention of DHM suggesting a positive effect of utilizing the DHM for psychological and mental conditions. A consistent reduction in body misestimation, except for height, was found indicating beneficial implications for people with AN (Keizer et al., 2016).

However, Keizer et al. (2016) did not find significant differences between synchronous and asynchronous visuotactile stimulation with DHM for body circumference estimation and body width estimation at height, hip, shoulder, and abdomen. Additionally, a nonsignificant improvement was found in the Hudson Index of Self-Esteem (p = 0.13), while the same study reported a significant improvement in self-esteem using the RSES (p = 0.02) (Nosek et al., 2016). Nonsignificant improvements in self-efficacy were also noted via the GSES (p = 0.08) (Nosek et al., 2016). Moreover, the PFRS and EPN-31 scales showed no significant changes in perceived fatigue or emotional improvement (p > 0.05) (Nuic et al., 2018). A nonsignificant decrease in EDI-DT and EDI-BU was found in both control and experimental groups, but a significant reduction in preoccupation with weight and fear of weight gain was observed after 1 month of DHM intervention. While the DDS-17 score showed significant improvement, no significant differences were found when comparing virtual DHM visits to in-person group visits among individuals with type II diabetes (p > 0.05) (Mitchell et al., 2023).

#### 3.5.4 Virtual experience-related outcome measures

Seven studies (Barhorst-Cates et al., 2022; Nuic et al., 2018; Kammler-Sücker et al., 2021; Nosek et al., 2016; Hall et al., 2011; Falconer et al., 2017; Keizer et al., 2016) evaluated the virtual experience using various assessment tools. The Embodiment Questionnaire (EQ) was commonly used in two studies (Barhorst-Cates et al., 2022; Keizer et al., 2016) to assess ownership, location, and agency. Keizer et al. (2016) (Keizer et al., 2016) found that both individuals with AN and healthy participants reported equally strong embodiment experience of DHM during training (p = 0.773). Furthermore, synchronous visuotactile stimulation using DHM significantly enhanced participants’ sense of ownership, agency, and spatial location compared to asynchronous stimulation (ownership: p < 0.001, location: p < 0.001, agency: p = 0.004) (Keizer et al., 2016). Similarly, Barhorst-Cate et al. (2022) (Barhorst-Cates et al., 2022) observed that individuals with left hemisphere stroke and healthy participants both reported equally moderate level of embodiment experience with DHM (p > 0.05). This experience was further evaluated across three different views of DHM—first-person view, anatomical view, and mirroring view, the results showed no significant differences among three views, however the first-person view yielded the highest, though not statistically significant (p > 0.05), level of embodiment experience in ownership, agency and spatial location over a DHM compared to the other two views (Barhorst-Cates et al., 2022). These findings highlighted the fact that the perspective used during DHM training does not largely affect the embodiment experience. Nevertheless, the moderate to high levels of embodiment reported underscore the potential usability of DHM in diverse rehabilitation training contexts.

Kammler-Sücker et al. (2021) used a modified version of the EQ - Autonomous avatar Question: positive avatar characteristics (AAQ1) assessing the embodiment experience of DHM. The findings showed that AAQ1 is significantly influenced by the type of avatar (pPB = 0.0010) (Kammler-Sücker et al., 2021). However, AAQ1 showed only a small to medium effect on the spinal lateral flexion range of motion (effect size βz = 0.1563; pSM = 0.0082, pPB = 0.0210) (Kammler-Sücker et al., 2021).

Self-designed surveys were used to evaluate virtual experiences in two studies (Nosek et al., 2016; Falconer et al., 2017). Nosek et al. (2016) reported a 3.16/4 score for the DHM system, indicating most of the participants rated the system as good and acceptable via their self-designed evaluation survey. Falconer et al. (2017) (Falconer et al., 2017) used a six-theme survey showing high acceptability for the DHM system in people with Parkinson’s disease (theme 1,2,6 scored 9/11, theme 4,5 scored 7/11, theme three scored 5/11).

Hall et al. (2011) reported high engagement (23–57 min), high accessibility (20/20), and cognitive presence (17/20), with lasting effects after 1 week. Nuic et al. (2018) found high feasibility using a Likert-scale questionnaire, with participants showing high perceived interest (p = 0.06), competence (p = 0.47), low difficulty (p = 0.87), and increased acceptability over time (p = 10⁻⁴).

#### 3.5.5 System-embedded parameters

Two studies (Xu et al., 2024; Kim et al., 2007) reported outcome measures that are embedded in the XR-based rehabilitation training system.

Kim et al. (2007) identified significant differences between individuals with neglect and healthy controls across parameters such as deviation angle, reaction time, visual cue, auditory cue, and mission failure rate, thereby confirming the system’s validity and usability in detecting neglect. Furthermore, the intervention included comparisons between the left and right eyes, revealing significantly poorer scores for the neglected eye in the embedded parameters. Additionally, the decrease in left-to-right ratio scores (representing asymmetry) indicated the neglect improved after treatment with DHM (p < 0.05) (Kim et al., 2007).

Another study by Xu et al. (2024) conducted a study assessing group-level event-related desynchronization (ERD), as well as peak ERD amplitude in the ipsilesional and contralesional hemispheres within the α and β frequency bands, to evaluate the effectiveness of synchronous and asynchronous visuotactile stimulation on a virtual body. The findings demonstrated that motor imagery from a third-person view with personalized DHM could enhance task performance in stroke patients (Xu et al., 2024). All parameters showed significant differences between synchronous and asynchronous stimulation (p < 0.05), therefore, highlighting the potential of virtual rehabilitation for stroke patients (Xu et al., 2024).

#### 3.5.6 Other outcome measures

Other types of outcome measures were also reported, such as medical outcomes study social support survey (MOS-SS), the relationship between imitation accuracy and limb apraxia measure, Mental rotation accuracy and reaction time, Block-mirroring accuracy and reaction time, Block-matching accuracy and reaction time were also reported in the studies (Barhorst-Cates et al., 2022; Nosek et al., 2016). The use of DHM revealed a non-significant improvement in emotional or informational support (Pre: 19.79 ± 6.47, Post:20.74 ± 5.83; t = −1.07, df = 18, p = 0.30) (Nosek et al., 2016).

While VR-based imitation tasks were not sensitive in detecting apraxia (χ2 (2) = 0.70, p = 0.402, DHM using anatomical, mirroring, and first-person views demonstrated potential cognitive enhancements, as the significant effects were presented in mental rotation, block-mirroring, and block-matching tasks (χ2 (2) = 6.32, p = 0.043, χ2 (2) = 8.96, p = 0.011, χ2 (2) = 8.96, p = 0.011) (Keizer et al., 2016).

## 4 Discussion

To the best of our knowledge, this is the first systematic review that comprehensively synthesizes the existing research on the effectiveness of 3D-4D DHM within XR-based rehabilitation. The findings across all included studies suggested that 3D DHM holds the potential to impact rehabilitation outcomes across several domains positively. These include functional outcomes, such as improved upper limb function (Tsekleves et al., 2016), gait performance (Nuic et al., 2018), balance function (Nuic et al., 2018), quality of life (Alemanno et al., 2019), and physical activity (Mitchell et al., 2023); physical outcomes, including decreased pain condition (Nuic et al., 2018; Alemanno et al., 2019), spasticity (Tsekleves et al., 2016), increased joint range of motion (Alemanno et al., 2019); psychological measures, such as improved depression and emotional regulation (Perpiñá et al., 2009; Nuic et al., 2018; Nosek et al., 2016; Falconer et al., 2017), reduced body image and weight concerns (Nosek et al., 2016; Keizer et al., 2016), and psychological impact (Mitchell et al., 2023); and general health outcomes, including improved body composition, and metabolic health (Mitchell et al., 2023; Sansoni et al., 2024). Moreover, when compared with conventional therapy, training involving 3D DHM showed largely greater improvements in eye movement of neglect patients (Kim et al., 2007), body image measures of anorexia nervosa patients (Perpiñá et al., 2009), eating disorder measures of bulimia nervosa patients (Sansoni et al., 2024), and diabetes-related indicators of type 2 diabetes patients (Faber and Fonseca, 2014). Additionally, 3D DHM was also demonstrated to be effectively utilized for detecting gait abnormalities (Booth et al., 2019; Zhu et al., 2022) and enhancing ownership of the embodiment experience (Barhorst-Cates et al., 2022; Keizer et al., 2016) during training, which highlights the potential as an innovative tool in XR-based rehabilitation. training.

However, unfortunately, we did not find any studies that related to the 4D DHM utilized directly by patients during treatment sessions, besides 3D DHM. Since 4D DHM advances beyond 3D DHM by enabling dynamic capture of the full body shape, incorporating both the 3D appearance (as in 3D DHM) and the temporal changes that occur during motor tasks (Meletani et al., 2024). All sixteen included studies showed positive results with the use of 3D DHM, therefore, we believe that 4D DHM could also offer added value by providing real-time, highly realistic, and dynamic visual feedback during training, which needs further investigation in future research. As the study by Meletani et al. (2024) proved the convincing reliability and accuracy in estimating gait spatiotemporal parameters and joint kinematics, it also highlighted the ability of the system to distinguish between healthy individuals and people with pathological conditions. Therefore, these huge advantages of 4D DHM should not be underestimated, particularly its accuracy and the informative data it provides, which open new frontiers for using DHM in improving clinical outcomes.

Although no 4D DHM was included in this review, it is important to highlight the findings of Kammler-Sücker et al. (2021), who demonstrated the greater similarity to the user’s realistic appearance in DHM, participants had better engagement and ownership of the embodiment in DHM while training, and thereby, highlighting the realism and personalization of the DHM could significantly affect ROM and movement performance (Kammler-Sücker et al., 2021).

DHM within the XR environment is considered a powerful tool for facilitating clinical outcomes and promoting self-correction during training (Liu et al., 2020; Barhorst-Cates et al., 2022). Nine studies focused on participants with neuromuscular diseases (Tsekleves et al., 2016; Kim et al., 2007; Booth et al., 2019; Barhorst-Cates et al., 2022; Zhu et al., 2022; Nuic et al., 2018; Kammler-Sücker et al., 2021; Alemanno et al., 2019), with four of these studies showing a preference for utilizing non–immersive VR combined with DHM in designed appearances (Tsekleves et al., 2016; Zhu et al., 2022; Nuic et al., 2018; Alemanno et al., 2019). This preference may arise from the increased risk of falls among participants, as fully immersive VR, although it enhances the level of illusion, can isolate users from their real environment, potentially compromising their ability to manage performance and maintain balance effectively. However, it is also notable that no incidences of falls were reported in either fully immersive or semi-immersive settings, as studies were predominantly conducted with participants in a seated position (Xu et al., 2024; Kim et al., 2007; Barhorst-Cates et al., 2022; Kammler-Sücker et al., 2021) or walking with dual belts (Booth et al., 2019) to ensure their safety. In contrast with neuromuscular patients, individuals with psychological, mental, or internal conditions do not often experience decreased balance function or impaired gait performance. However, five (Mitchell et al., 2023; Perpiñá et al., 2009; Nosek et al., 2016; Hall et al., 2011; Falconer et al., 2017) out of seven studies in these populations were still conducted within a non-immersive VR environment on a computer, which might be caused by the concern about the accessibility and ecology of the training itself. Furthermore, only one study from Sansoni et al. (2024) was performed with a DHM in high-realism appearances and well-designed animation in an AR setting. Nevertheless, no significant different effects were found between AR (Sansoni et al., 2024) and VR (Perpiñá et al., 2009) as shown in Supplementary Table S1, due to lack of comparable parameters. In conclusion, despite the moderate to high risk of bias presented in the included studies, the majority (Mitchell et al., 2023; Perpiñá et al., 2009; Tsekleves et al., 2016; Zhu et al., 2022; Nuic et al., 2018; Nosek et al., 2016; Alemanno et al., 2019; Hall et al., 2011; Falconer et al., 2017) demonstrated a preference for utilizing non-immersive VR settings in combination with DHM for rehabilitation training.

Given that visual feedback is a crucial element in influencing performance and maintaining balance in individuals with neurological conditions (Chen et al., 2021), thus, the patient’s performance could vary depending on the different visual content. Further investigations were conducted. Barhorst-Cates et al. (2022) found that using a first-person view of DHM yielded better outcomes and higher ownership of the DHM, although the differences between the first-person view, mirroring view, and anatomical view were not statistically significant. This lack of significant difference might be attributed to the relatively low difficulty of the tasks involved. Xu et al. (2024) explored the third-person view of DHM and found that it generated a strong sense of ownership among stroke patients, though this study lacked a comparison group with an alternative view. Considering the level of evidence, both two studies bring moderate risk of bias to this review, whereas Xu et al. (2024) obtained an EBRO level as an A2 and Barhorst-Cates et al. (2022) obtained a B. Consequently, we agree that a third-person view of DHM could elicit clinical results by inducing a stronger sense of ownership. However, non-immersive VR with a first-person view of DHM was also able to enhance better clinical outcomes in training with DHM (Barhorst-Cates et al., 2022). The results of the EQ in the study by Barhorst-Cates et al. (2022) indicated higher ownership scores with the first-person view, suggesting that this point of view of DHM may better align with participants’ perceptions of their own bodies. This finding is consistent with previous studies from Slater et al. (2009), Slater et al. (2008), Petkova et al. (2011). Therefore, to further clarify the outcomes of these two perspectives, fuure research should address the comparative effectiveness of first-person *versus* third-person views of DHM to determine which method offers the most significant clinical benefits.

Moreover, two studies (Xu et al., 2024; Keizer et al., 2016) further compared the synchronous and asynchronous visuo-tactile stimulation of the actual and virtual body (DHM). Xu et al. (2024) reported that both synchronous and asynchronous visuo-tactile enhanced brain activation, however, the synchronous visuo-tactile stimulation led to stronger brain activation compared to the asynchronous condition. This was accompanied by an increased event-related desynchronization amplitude in the α and β frequency bands (Xu et al., 2024), which enhanced motor neuron activity, potentially improving motor function restoration and aiding movement initiation ability in stroke patients during motor imagery enhancement (77, 78). While the findings of Keizer et al. (2016) align with those of Xu et al. (2024), demonstrating a positive effect of DHM in both synchronous and asynchronous visuotactile stimulations, with a significant reduction in the misestimation of all body circumferences except for height and abdomen width (Keizer et al., 2016). Participants reported more embodiment of DHM with synchronous stimulation compared to asynchronous stimulation, however, did not show significantly larger amounts of changes in body size estimation between two stimulations, which indicated that the synchronicity of the visuotactile stimulation is not important for changing the experience of body size (Keizer et al., 2016). In short, DHM itself could potentially enhance brain activation, boost motor restoration, and decrease the misestimation of body circumferences. The DHM with synchronous visuo-tactile stimulation could further improve better embodiment of the DHM, compared with asynchronous visuo-tactile stimulation.

Another interesting remark is that while most of the results indicated the positive effectiveness of DHM, its role in interventions for psychological conditions might differ from its role in neurorehabilitation. In psychological and mental health interventions, DHM primarily helps people with anorexia nervosa (Perpiñá et al., 2009; Keizer et al., 2016), bulimia nervosa (Perpiñá et al., 2009; Sansoni et al., 2024), borderline personality disorder (Falconer et al., 2017), and intellectual disabilities (Hall et al., 2011) by addressing the multisensory processing deficit, changing distorted body image perception, and using the perspective-taking function, encouraging participants to engage and reflect more deeply with their emotional feelings and cognitive experiences, thereby enhancing self-awareness to facilitate outcomes (Falconer et al., 2017; Sansoni et al., 2024; Brizzi et al., 2023). In contrast, neurorehabilitation and neuromuscular rehabilitation for people with neglect (Kim et al., 2007), hemispheric disease (Tsekleves et al., 2016; Barhorst-Cates et al., 2022; Zhu et al., 2022), LBP (Kammler-Sücker et al., 2021; Alemanno et al., 2019), PD (Nuic et al., 2018), CP (Booth et al., 2019), and dyskinesia (Xu et al., 2024), DHM primarily functions as a real-time imitation and simulation tool for movement and performance feedback, allowing users to enhance the quality of their performance through self-correction (Liu et al., 2020; Escarti and Guzman, 1999). In this way, DHM helps patients regain control over their physical movements by fostering a more active and engaged learning process within the brain, particularly in cases where motor functions are impaired (Xu et al., 2024). Overall, DHM can provide different effects to enhance self-awareness, self-recognition, and self-correction over various pathologies in the rehabilitation process.

As the final consideration, only one study by Mitchell et al. (2023), utilized 3D DHM in XR environment on a large scale, involving three hundred type 2 diabetes patients. The findings demonstrated that DHM home telerehabilitation achieved clinical outcomes comparable to in-person care in controlling hemoglobin A1c levels, increasing physical activity, and enhancing patient engagement. Despite this RCT study having a moderate risk of bias, the study results highlighted the potential benefits of DHM, including reduced therapist workload and increased treatment portability for patients. In contrast, the remaining studies included in this review were conducted on no more than 30 patients (Xu et al., 2024; Perpiñá et al., 2009; Tsekleves et al., 2016; Kim et al., 2007; Booth et al., 2019; Barhorst-Cates et al., 2022; Zhu et al., 2022; Nuic et al., 2018; Kammler-Sücker et al., 2021; Nosek et al., 2016; Alemanno et al., 2019; Hall et al., 2011; Falconer et al., 2017; Sansoni et al., 2024; Keizer et al., 2016). Four studies (Xu et al., 2024; Barhorst-Cates et al., 2022; Kammler-Sücker et al., 2021; Keizer et al., 2016) explored the mechanisms underlying DHM rehabilitation benefits. For instance, stroke patients showed improved voluntary imitation (Kammler-Sücker et al., 2021) and accuracy (Barhorst-Cates et al., 2022), as well as enhanced motor imagery (Xu et al., 2024), while AN patients experienced improvements in body image disturbance (Keizer et al., 2016). The other eleven studies (Perpiñá et al., 2009; Tsekleves et al., 2016; Kim et al., 2007; Booth et al., 2019; Zhu et al., 2022; Nuic et al., 2018; Nosek et al., 2016; Alemanno et al., 2019; Hall et al., 2011; Falconer et al., 2017; Sansoni et al., 2024) focused on the validation and feasibility of using DHM in rehabilitation, yielding promising outcomes but highlighting significant limitations, such as DHM has not yet been widely adopted in patients’ daily routine training programs. Most applications remain experimental and are not accessible to the general public, likely due to technical complexity, high costs, and the absence of standardized training protocols. Addressing these barriers is critical for the successful clinical translation of DHM technology.

Regarding the limitations of this systematic review, the level of included studies was relatively low with only one RCT and one cohort study. The remaining consisted of five non-randomized case-control studies, six pre-post-experimental studies, and two case studies without comparison groups. Therefore, the lack of robust control groups or randomized designs might introduce bias and confounders into the results. Secondly, it should be noted that the sample sizes were also significantly insufficient in some studies, potentially limiting the generalizability of the findings and validity of the studies (Faber and Fonseca, 2014). While four studies had a large sample size population of more than fifty participants (Mitchell et al., 2023; Kim et al., 2007; Booth et al., 2019; Keizer et al., 2016), only Keizer et al. (2016) achieved a low-risk bias, with the others offering a moderate risk of bias for this review. In addition, besides Keizer et al. (2016), the overall risk of bias in all included studies ranged from moderate to high. Besides Xu et al. (2024) obtained a level of A2, the rest of the EBRO levels ranged from level B-level C. Apart from Xu et al. (2024), which was rated at level A2, the rest of the EBRO levels ranged from level B to level C. Consequently, these fair to low-quality studies may weaken the strength and reliability of the conclusions and diminish the statistical power of the review. Thirdly, the search strategy was restricted to English-language studies, and therefore, would result in publication bias and reduce the statistical power of the overall estimations.

Finally, the interventions and outcome measures used in the included studies showed considerable variations and combinations, combined with the limited number of comparable eligible studies, which made it difficult to conduct a meta-analysis for this systematic review. These variations of the outcome measures might be due to a lack of standardized designs for rehabilitation training and, therefore, complicate the comparison and interpretation of the results from the included studies. Despite these challenges, most findings of the studies were aligned with each other, highlighting the usability and positive effectiveness of DHM in assessment and training. This systematic review pointed out the urgent need for standardized designs or protocols in XR-based DHM rehabilitation settings to improve future research quality.

To address the limitations identified in this review, future research should focus on several points. Firstly, high-quality RCTs with a larger scale of participants are necessary, not only to increase the strength of the study but also to establish reliable results regarding the efficacy of 3D-4D DHM in XR-based rehabilitation. Secondly, future studies should add comparative groups with conventional treatment to reveal a clear understanding of the true added value of the interventions. Thirdly, future studies must develop standardized protocols to address the variability in study parameters within the same domain. This lack of standardization also explains why no studies utilizing 4D DHM could be included in this review because of the variety and non-comparable parameters.

In addition to the focus as described above, from a technological perspective, it is also essential to address the complexity of developing 4D DHM rehabilitation training systems to facilitate practical transition in clinical settings. To overcome this barrier, we suppose that close collaboration between rehabilitation therapists and computer science engineers is very critical for advancing application development. Such an interdisciplinary approach is important for filling the gap between technology superiority and practical transition to develop user-friendly and clinically applicable applications.

Finite element (FE) joint modeling could also serve as a complementary solution to address this issue. A FE model of the entire knee joint was developed and extensively investigated by Adouni et al. (2019), Adouni et al. (2020), their findings demonstrated the model’s capability to simultaneously predict macro-continuum joint mechanics, including compressive forces and stress distribution. Further validation by Faisal et al. (2019) confirmed the model’s ability to detect and record the mechanical response of cartilage in the knee, presenting its potential for understanding complex biomechanical behaviors in joint structures while movement.

Building upon these capabilities of the FE joint modeling, integrating 4D DHM with this technology could provide critical insights into dynamic movements and biomechanical analysis, serving as a complementary solution to address the current challenges in this new field. This combined approach has the potential to significantly enhance the precision, personalization, and overall effectiveness of rehabilitation interventions. Furthermore, it could strengthen the clinical relevance and applicability of 4D DHM, paving the way for its broader integration into rehabilitation training programs.

The implications of this systematic review are significant for the future of XR-based rehabilitation, particularly in integrating 3D and 4D DHM. The consistently positive results from 3D DHM across various patient groups indicate that this technology can enhance rehabilitation outcomes, especially when compared to conventional therapies. The potential of 4D DHM, which captures dynamic body movements, suggests that it could offer even greater benefits, though this requires further investigation. Despite the current lack of direct application studies for 4D DHM and some technological limitations, its accuracy and detailed feedback capabilities could open new avenues in clinical practice. However, the limitations of the included studies, such as small sample sizes and potential biases, highlight the need for more rigorous research to strengthen these findings and support the broader adoption of DHM in rehabilitation.

## 5 Conclusion

In conclusion, this review is the first to systematically analyze the effectiveness of 3D-4D DHM in XR-based rehabilitation, revealing consistently positive outcomes and sensitive assessments with 3D DHM across various patient groups. Although 4D DHM was still undiscovered, its potential for enhancing real-time feedback and improving clinical results is promising and warrants further research. Despite limitations, such as small sample sizes and moderate to high risks of bias, the findings suggest that DHM, particularly in non-immersive VR settings, could be a valuable tool in rehabilitation. Future studies should focus on optimizing DHM’s application, particularly comparing first-person and third-person views, and exploring 4D DHM’s full potential.

## Data Availability

The original contributions presented in the study are included in the article/Supplementary Material, further inquiries can be directed to the corresponding author.
