# Supplementary material for: Using 3D and 4D digital human modeling in extended reality-based rehabilitation: a systematic review
Source: Front Bioeng Biotechnol. 2025 Mar 12;13:1496168. doi: 10.3389/fbioe.2025.1496168 (PMC11937100; doi:10.3389/fbioe.2025.1496168)
Supplement: Supplementary file 1 [file Table1.docx]

**Appendix 1. Search Strategy**

| **Database** | **Full search strategy** | **Number of hits** |
| --- | --- | --- |
| PubMed | ("Patients"[MeSH Terms] OR patient OR patients) AND ( “4D Digital Human Model*” OR “3D Digital Human Model*” OR "Somatotypes"[Mesh] OR "Body Surface Area"[Mesh] OR "Body Image"[Mesh] OR "Body shape" OR humanlike OR “Personal avatar” OR chatbot OR “Digital Twin” OR "Body surface" OR "Body Silhouette" OR "Body mesh" OR "Body representation") AND ("Virtual Reality"[MeSH Terms] OR "Virtual Reality" OR VR OR " Virtual Human" OR "Virtual Patient Modeling" OR "Telerehabilitation"[MeSH Terms] OR Telerehabilitation OR "Telemedicine"[MeSH Terms] OR "Mobile Health Units"[MeSH Terms] OR "Mobile health" OR mHealth OR "Augmented Reality"[MeSH Terms] OR "Extended reality" OR "Augmented Reality" OR "Mixed Reality" OR "Gamification"[MeSH Terms] OR gamification OR "Internet of Things"[MeSH Terms] OR "internet of things") AND ("Postural Balance"[MeSH Terms] OR "Posture"[MeSH Terms] OR Performance OR Balance OR Posture OR "Gait"[MeSH Terms] OR "Education"[MeSH Terms] OR "Education"[MeSH Subheading] OR education OR "Awareness"[MeSH Terms] OR "Ergonomics"[MeSH Terms] OR "Biomechanical Phenomena"[MeSH Terms] OR biomechanics OR "Treatment Outcome"[MeSH Terms] OR Effectiveness OR Reliability) | 427 |
| IEEE | ("All Metadata":"patient" OR "All Metadata":"patients") AND ("All Metadata":"4D Digital Human Model*" OR "All Metadata":"3D Digital Human Model*" OR "All Metadata":"Somatotypes" OR "All Metadata":"Body Surface Area" OR "All Metadata":"Body Image" OR "All Metadata":"Body shape" OR "All Metadata":"humanlike" OR "All Metadata":"Personal avatar" OR "All Metadata":“Chatbot” OR "All Metadata":"Digital Twin" OR "All Metadata":"Body surface" OR "All Metadata":"Body Silhouette" OR "All Metadata":"Body mesh" OR "All Metadata":"Body representation") AND ("All Metadata":"Virtual Reality" OR "All Metadata":"VR" OR "All Metadata":"Virtual Human" OR "All Metadata":"Virtual Patient Modeling" OR "All Metadata":"Telerehabilitation" OR "All Metadata":"Mobile health" OR "All Metadata":"mHealth" OR "All Metadata":"Augmented Reality" OR "All Metadata":"Extended reality" OR "All Metadata":"Mixed Reality" OR "All Metadata":"Gamification" OR "All Metadata":"Internet of Things") AND ("All Metadata":"Postural Balance" OR "All Metadata":"Performance" OR "All Metadata":"Balance" OR "All Metadata":"Posture" OR "All Metadata":"Gait" OR "All Metadata":"Education" OR "All Metadata":"Awareness" OR "All Metadata":"Ergonomics" OR "All Metadata":"Biomechanical Phenomena" OR "All Metadata":"biomechanics" OR "All Metadata":"Treatment Outcome" OR "All Metadata":"Effectiveness" OR "All Metadata":"Reliability") | 76 |
| Cochrane Library | ("Patients" OR "patient" ) AND (4D Digital Human Model* OR 3D Digital Human Model* OR "Somatotypes" OR "Body Surface Area" OR "Body Image" OR "Body shape" OR "humanlike" OR "Personal avatar" OR "Digital Twin" OR "Body surface" OR "Body Silhouette" OR "Body mesh" OR "Body representation") AND ("Virtual Reality" OR "VR" OR "Virtual Human" OR "Virtual Patient Modeling" OR "Telerehabilitation" OR "Mobile health" OR "mHealth" OR "Augmented Reality" OR "Extended reality" OR "Mixed Reality" OR "Gamification" OR "Internet of Things" ) AND ("Postural Balance" OR "Posture" OR "Performance" OR "Balance" OR "Gait" OR "Education" OR "Awareness" OR "Ergonomics" OR "Biomechanical Phenomena" OR "biomechanics" OR "Treatment Outcome" OR "Effectiveness" OR "Reliability") | 97 |
| Web of Science | ((((ALL=("patient" OR "patients")) AND ALL=("4D Digital Human Model*" OR "3D Digital Human Model*" OR "Somatotypes" OR "Body Surface Area" OR "Body Image" OR "Body shape" OR "humanlike" OR "Personal avatar" OR “Chatbot” OR "Digital Twin" OR "Body surface" OR "Body Silhouette" OR "Body mesh" OR "Body representation")) AND ALL=("Virtual Reality" OR "VR" OR "Virtual Human" OR "Virtual Patient Modeling" OR "Telerehabilitation" OR "Mobile health" OR "mHealth" OR "Augmented Reality" OR "Extended reality" OR "Mixed Reality" OR "Gamification" OR "Internet of Things")) AND ALL=("Postural Balance" OR "Performance" OR "Balance" OR "Posture" OR "Gait" OR "Education" OR "Awareness" OR "Ergonomics" OR "Biomechanical Phenomena" OR "biomechanics" OR "Treatment Outcome" OR "Effectiveness" OR "Reliability")) | 156 |
| Science Direct | (Patient) AND (4D Digital Human Model OR 3D Digital Human Model) AND ("Virtual Reality" OR "Telerehabilitation" OR Gamification) AND ("Posture" OR "Ergonomics" OR "biomechanics")  Filter: Research articles | 292 |
