# Supplementary material for: Using 3D and 4D digital human modeling in extended reality-based rehabilitation: a systematic review
Source: Front Bioeng Biotechnol. 2025 Mar 12;13:1496168. doi: 10.3389/fbioe.2025.1496168 (PMC11937100; doi:10.3389/fbioe.2025.1496168)
Supplement: Supplementary file 2 [file Table2.docx]

**Supplementary Table S1.** Table of outcome measures, results, and interpretations

| **Reference** | **Outcome Measures** | **Significant results  => Interpretation** |
| --- | --- | --- |
| C.Perpina et al. (1999) | **Psychological:** Both CG and EG improved in all measures after treatment | |
|  | Beck Depression Inventory | F(1,10)=4.85, p<0.05 **=>** EG larger improvement |
|  | Positive and Negative Affect Schedule | F(1,10)=6.28, p<0.03 **=>** EG larger improvement |
|  | Body Areas Satisfaction Scale | F(1,10)=10.5, p<0.009 **=>** EG more satisfied |
|  | Situational Inventory of Body Image Dysphoria | F(1,10)=16.57, p<0.002 **=>** EG felt less dysphoria in showing their body |
|  | Body Image Avoidance Questionnaire | F(1,10)=5.27, p<0.04 **=>** EG less body avoidance |
|  | Body Image Automatic Thoughts Questionnaire | F(1,10)=12.04, p<0.006 **=>** EG less negative thoughts for BI |
|  | Fear of putting on weight | F(1,10)=6.28, p<0.03 **=>** EG felt less fear or putting on weight |
|  | Body Shape Questionnaire | F(1,10)=30.8, p<0.0001 **=>** EG showed more satisfaction with the body |

**Table 3b.** Cont.

| Kim et al. (2007) | **System embedded parameters:** Compare to CG1: No difference between CG1 & CG2 | |
| --- | --- | --- |
|  | Deviation angle | EG=-9.17±4.37, CG1=-0,36±1.18, p<0.05 **=>** EG deviate to left |
|  | Reaction time (sec) | EG=7.77±1.84, CG1=4.94±1.07, p<0.05 **=>** EG react slower |
|  | Visual cue (%) | EG=45.28±27.81, CG1=2.70±5.29, p<0.05 **=>** EG more visual cue |
|  | Auditory cue (%) | EG=31.65±34.12, CG1=0.00±0.00, p<0.05 **=>** EG more auditory cue |
|  | Failure rate of mission (%) | EG=8.65±10.12, CG1=0.00±0.00, p<0.05 **=>** EG higher failure rate |
|  | **Compare between right & left side:** No difference between CG1 right eye & CG1 left eye | |
|  | Reaction time (sec) | EG R=6.09±1.22, EG L=8.63±2.29, p<0.05 **=>** EG left eye react slower |
|  | Visual cue (%) | EG R=16.67±17.06, EG L=63.26±19.31, p<0.05 **=>** EG left eye more visual cue |
|  | Auditory cue (%) | EG R=1.00±3.16, EG L=46.12±45.61, p<0.05 **=>** EG left eye more auditory cue |
|  | Failure rate of mission (%) | EG R=0.00±0.00, EG L=12.14±13.62, p<0.05 **=>** EG left eye higher failure rate |
|  | Left-to-right ratio scores  (representing asymmetry) | Score ↓ **=>** EG left and right asymmetry ↓  **=>** neglect ↑ |

**Table 3b.** Cont.

| Hall et al. (2011) | **Virtual experience** | |
| --- | --- | --- |
|  | Temporality (voluntary stay duration) | 23min-57min **=>** Most of participants remained 40min-45min voluntarily |
|  | Accessibility (move avatar) | 20/20 participants **=>** All participants are able to finish the treatment without any physical difficulty |
|  | Context | 4/20 participant **=>** Only a few participants looked similar to their avatar |
|  | Cognitive presence | 17/20 participants **=>** Most of participants show identification of the scenario |
|  | Recall (after 1 week) | 20/20 participants **=>** All participants are able to recall some accurate memories |
| Tsekleves et al. (2016) | **Functional** | |
|  | FMA (upper limb section) | ↑ from 39 to 47 **=>** Significant ↑ in upper limb impairment |
|  | NHPT | ↓ from 29 sec per peg to 7.8 sec per peg (from two drops to no drop) **=>** Significant ↑ in upper limb impairment |
|  | MAL (AOU sub-scale) | ↑ from 7 to 10 **=>** Significant ↑ in functional spontaneous use of the affected arm |
|  | **Physical** | |
|  | MAS (shoulder and elbow) | Score unchanged **=>** Remained unchanged spasticity in shoulder and elbow |
|  | MAS (finger and wrist) | ↓ from 3 to 1 **=>** A non-significant reduction in spasticity of finger and wrist flexors |

**Table 3b.** Cont.

| Keizer et al. (2016) | **Psychological** | |
| --- | --- | --- |
|  | BW estimation: Height (Syn & Asyn) | F(2,56)=1.89, p=0.160, η2=0.06 **=>** Estimation height unchanged;  **=>** No sig. difference between Syn & Asyn |
|  |  | F(1,57)=0.05, p=0.830, η2=0.00 **=>** No sig. difference between EG & CG |
|  | BW estimation: Shoulder (Syn & Asyn) | F(2,56)=14.16, p<0.001, η2=0.34 **=>** Misestimation shoulder ↓ pre & post;  **=>** No sig. difference between Syn & Asyn |
|  |  | F(1,57)=23.50, p<0.001, η2=0.29 **=>** Misestimation ↓ more in EG |
|  | BW estimation: Abdomen (Syn & Asyn) | F(2,56)=0.15, p=0.860, η2=0.01 **=>** No sig. ↓ pre & post;  **=>** No sig. difference between Syn & Asyn |
|  |  | F(1,57)=25.61, p<0.001, η2=0.31 **=>** Misestimation ↓ more in EG |
|  | BW estimation: Hip (Syn & Asyn) | F(2,56)=4.10, p=0.022, η2=0.13 **=>** Misestimation hip ↓ pre & post in Syn;  **=>** No sig. ↓ pre & post in Asyn |
|  |  | F(1,57)=24.56, p<0.001, η2=0.3 Misestimation ↓ more in EG |

**Table 3b.** Cont.

| Keizer et al. (2016) | BC estimation: Shoulder (Syn & Asyn) | F(2,56)=23.83, p<0.001, η2=0.46 **=>** Misestimation shoulder ↓ pre & post;  **=>** No sig. difference between Syn & Asyn |
| --- | --- | --- |
|  |  | F(1,57)=33.57, p<0.001, η2=0.37 **=>** Misestimation ↓ more in EG |
|  | BC estimation: Abdomen (Syn & Asyn) | F(2,56)=4.23, p=0.19, η2=0.13 **=>** EG misestimation abdomen ↓ pre & post;  **=>** CG no sig. difference pre & post |
|  |  | F(1,57)=42.27, p<0.001, η2=0.43 **=>** Misestimation ↓ more in EG;  **=>** No sig. difference between Syn & Asyn |
|  | BC estimation: Hip (Syn & Asyn) | F(2,56)=24.26, p<0.001, η2=0.46 **=>** Misestimation hip ↓ pre & post;  **=>** No sig. difference between Syn & Asyn |
|  |  | F(1,57)=42.14, p<0.001, η2=0.43 **=>** Misestimation ↓ more in EG |
|  | **Virtual experience** | |
|  | Syn & Asyn: | F(1,57)=0.08, p=0.773, η2=0.00 **=>** No sig. difference between the experience of the FBI in EG & CG. |
|  | EQ: Ownership | t(58)=1.17, p<0.001, d=0.60 **=>** More ownership of FBI in Syn compared to Asyn |
|  | EQ: Location | t(58)=1.88, p<0.001, d=1.02 **=>** More location of FBI in Syn compared to Asyn |

**Table 3b.** Cont.

| Keizer et al. (2016) | EQ: Agency | t(58)=1.90, p=0.004, d=0.40 More agency of FBI in Syn compared to Asyn |
| --- | --- | --- |
| Nosek et al. (2016) | **Psychological** | |
|  | Depression:  Center for Epidemiologic Studies Depression scale-10 | ↓ from 11.44±6.36 to 7.81±3.38; t=3.21, df=18, p=0.005, Cohen’s d=0.74 **=>** Significant ↑ in depression, moderate to large ES |
|  | Self-Esteem:  Rosenberg Self-Esteem scale | ↑ from 19.84±5.75 to 22.21±4.87; t=-2.62, df=18, p=0.02, Cohen’s d=0.60 **=>** Significant ↑ in self-esteem, moderate ES |
|  | Self-Esteem:  Hudson Index of Self-Esteem | ↑ from 67.32±18.95 to 71.13±16.27; t=-1.59, df=18, p=0.13, Cohen’s d=0.36 **=>** No sig. ↑, small to moderate ES |
|  | Self-Efficacy: Generalized Self-Efficacy scale | ↑ from 29.79±5.70 to 31.53±4.29; t=-1.84, df=18, p=0.08, Cohen’s d=0.42 **=>** No sig. ↑, small to moderate ES |
|  | **Virtual experience** | |
|  | Acceptability: Self-designed Evaluation Survey | Excellent/Good (83.3%) overall score 3.16/4 **=>** Most of the participants rated the intervention as good and acceptable |
|  | **Other** | |
|  | Social Support: Medical Outcomes Study Social Support Survey | ↑ from 19.79±6.47 to 20.74±5.83; t=-1.07, df=18, p=0.30, Cohen’s d=0.25 **=>** No sig. ↑, small to moderate ES |

**Table 3b.** Cont.

| Falconer et al. (2017) | **Psychological** | |
| --- | --- | --- |
|  | 21-item Depression, Anxiety and Stress Scales | smallest p=0.12 **=>** No sig. changes were found across four sessions |
|  | Mentalization Questionnaire | smallest p=0.12 **=>** No sig. changes were found across four sessions |
|  | **Virtual experience** | |
|  | Acceptability: Self-designed six-theme Survey | Theme 1: Yes (9/11); Theme 2: Yes (9/11); Theme 3: Yes (5/11);  Theme 4: Yes (7/11); Theme 5: Yes (7/11); Theme 6: Yes (9/11). **=>** Positive effect of avatar-MBT, gaining fresh insights into daily-life situations. |
| Nuic et al. (2018) | **Psychological** | |
|  | Piper Fatigue Revised Scale | 3.77±1.79; χ2 [5] =3.893, p=0.56 **=>** No sig. change in perceived fatigue |
|  | Negative Emotionality questionnaire (EPN-31) | 42.85±16.66; χ2[5]=3.084, p=0.69 **=>** No sig. change in negative emotion |
|  | Positive Emotionality questionnaire (EPN-31) | 42.3±11.2; χ2[5,45]=1.879, p=0.11 **=>** No sig. ↓ in positive emotion |
|  | **Functional** | |
|  | UPDRS part III | ↓ (p=0.34) **=>** No sig. change between pre & post in motor Parkinsonian disability |
|  | UPDRS part II  (Activities of daily living) | ↓ (p=0.03) **=>** Sig. ↑ between pre & post in activities of daily living |

**Table 3b.** Cont.

| Nuic et al. (2018) | PDQ-39 | ↓ (p=0.13) **=>** No sig. change in quality of life |
| --- | --- | --- |
|  | Axial score (UPDRS items 18 +27 +28+29+30) | ↓ 41% (p=0.009) **=>** No sig. difference between Syn & Asyn;  **=>** Sig. ↑ in gait and balance |
|  | Freezing of Gait Questionnaire | ↓ 39% (p=0.005) **=>** Sig. ↑ in freezing of gait |
|  | Activities and Balance Confidence scale | ↑ 35% (p=0.03) **=>** Sig. ↑ in Parkinsonian disability |
|  | Gait and Balance Scale Part B | ↓ 38% (p=0.02) **=>** Sig. ↑ in gait and balance |
|  | Gait parameters: APAs | ↓ (p=0.0004) **=>** Sig. ↓ APAs phase |
|  | Gait parameters: Double stance durations | ↓ (p=0.0002)  **=>** Sig. ↓ double stance durations |
|  | Gait parameters: Anteroposterior APAs displacement | ↑ (p<10-4) **=>** Sig. ↑ anteroposterior APAs displacement |
|  | Gait parameters: Step length | ↑ (p <10-4) **=>** Sig. ↑ step length |
|  | Gait parameters: Gait velocity | ↑ (p <10-4) **=>** Sig. ↑ gait velocity |

**Table 3b.** Cont.

| Nuic et al. (2018) | Gait parameters:  Mediolateral APAs displacement | (p>0.05) **=>** No sig. change |
| --- | --- | --- |
|  | Gait parameters:  Step width |  |
|  | Gait parameters:  Braking Index |  |
|  | **3-month follow-up** | |
|  | Gait parameters: Anteroposterior APAs, Step length, Gait velocity | ↑ (p<0.05) **=>** Sig. ↑ in gait function 3 months later |
|  | Axial score (UPDRS items 18 +27 +28+29+30) | ↓ 35% (p=0.005) **=>** Sig. ↑ in gait and balance 3 months later |
|  | **Virtual experience** | |
|  | Feasibility: LSSQ:  perceived interest | 25.4± 3.4; χ2[5]=10.6, p=0.06 **=>** High-perceived interest and stable over time |
|  | Feasibility: LSSQ:  perceived competence | 41.3±9.1; F[5, 45]=0.92, p=0.47 **=>** High-perceived competence and stable over time |
|  | Feasibility: LSSQ:  perceived difficulty | 11.85±3.4; F[5, 45]=0.36, p=0.87 **=>** Low-perceived difficulty and stable over time |
|  | Acceptability scale | 8.39±1.56; χ2[5]=20.5, p=10-4 **=>** General acceptability sig. ↑ over time |

**Table 3b.** Cont.

| Alemanno et al. (2019) | **Physical** | |
| --- | --- | --- |
|  | NRS pain scale | ↓ 4.5 (p<0.001) **=>** Sig. ↓ pain after treatment |
|  | the McGill pain Questionnaire | ↓ 8.5 (p=0.001) **=>** Sig. ↓ pain after treatment |
|  | Brief pain Inventory | ↓ 24.66 (p<0.001) **=>** Sig. ↓ pain after treatment |
|  | Number of Words Chosen | ↓ 3.55 (p=0.001) **=>** Sig. ↓ pain after treatment |
|  | Pain score at worst | ↓ 19.44 (p=0.002) **=>** Sig. ↓ pain after treatment |
|  | Average pain score | ↓ 20 (p<0.001) **=>** Sig. ↓ pain after treatment |
|  | Global Impression of Change | 16/20; 3/20 **=>** 16 patients ↑;  **=>** 3 patients no change |
|  | Trunk ROM: maximal rotation | ↑ 16.47 (p=0.002)  **=>** sig. ↑ ROM |
|  | Trunk ROM: average rotation | ↑ 19.65 (p=0.008) **=>** sig. ↑ ROM |
|  | Kinematic data: repetition index | ↑ 0.15 (p=0.024)  **=>** sig. ↑ in proprioception |

**Table 3b.** Cont.

| Alemanno | **Psychological** | |
| --- | --- | --- |
| et al. (2019) | Beck Depression Inventory-II | ↓ 10 (p=0.037) **=>** Sig. ↑ |
|  | Neuropsychological evaluations: cognition | Sig. ↑ (p<0.05) **=>** No sig. abnormalities in cognition |
|  | **Functional** | |
|  | SF-36 | (p<0.05) **=>** 5/8 subscale score sig. ↑ |
|  | The Roland and Morris Disability Questionnaire | ↓ 5.79 (p<0.001) **=>** Sig. ↑ in participation |
| Booth et al. (2019) | **Physical** | |
|  | Mean pelvis tilt | CG: HBM: 10.9±5.9; 8MM: 10.9±5.8 (p=0.880); EG: HBM: 17.0±5.1; 8MM: 17.9±5.3 (p=0.009). **=>** Sig. ↑ in 8MM;  **=>** No sig. difference between HBM & 8MM |
|  | Pelvic ROM: tilt | CG: HBM: 4.6±1.0; 8MM: 4.8±1.0 (p=0.167); EG: HBM: 7.0±2.4; 8MM: 6.4±2.1 (p=0.01). **=>** Sig. ↓ in 8MM;  **=>** No sig. difference between HBM & 8MM |
|  | Pelvic ROM: obliquity | CG: HBM: 11.2±2.4; 8MM: 8.5±2.1 (p<0.001); EG: HBM: 9.6±3.3; 8MM: 6.6±2.3 (p<0.001). **=>** Sig. slight ↓ in 8MM |

**Table 3b.** Cont.

| Booth et al. (2019) | Pelvic ROM: rotation | CG: HBM: 11.1±3.3; 8MM: 10.8±3.0 (p=0.315); EG: HBM: 13.6±3.8; 8MM: 13.2±3.9 (p=0.427). **=>** No sig. difference between HBM & 8MM |
| --- | --- | --- |
|  | Hip ROM: flexion | CG: HBM: 43.1±5.5; 8MM: 40.9±5.0 (p<0.001); EG: HBM: 39.9±7.5; 8MM: 37.1±7.1 (p<0.001). **=>** Sig. ↓ in 8MM |
|  | Knee ROM: flexion | CG: HBM: 65.4±6.0; 8MM: 52.4±5.2 (p<0.001); EG: HBM: 47.7±11.9; 8MM: 41.1±9.5 (p<0.001). **=>** Sig. ↓ in 8MM |
|  | **Functional** | |
|  | Step length | CG: HBM: 0.50±0.09; 8MM: 0.50±0.08 (p=0.740); EG: HBM: 0.37±0.09; 8MM: 0.36±0.09 (p=0.798). **=>** No sig. difference between HBM & 8MM |
|  | Step width | CG: HBM: 0.22±0.03; 8MM: 0.22±0.03 (p<0.001); EG: HBM: 0.27±0.06; 8MM: 0.26±0.06 (p<0.001). **=>** Sig. ↓ in 8MM, yet negligible. |
|  | Cadence | CG: HBM: 116±16; 8MM: 119±13 (p=0.176); EG: HBM: 100±13; 8MM: 104±21 (p=0.412). **=>** No sig. difference between HBM & 8MM |

**Table 3b.** Cont.

| Kammler-Sucker et al. (2021) | **Physical:** (Compare Avatar 1, 2, 3 and 4) | |
| --- | --- | --- |
|  | Spinal functional ROM: LF | β=0.0619, pSM=0.0771, pPB=0.0975 **=>** Sig. ↑ indicate a linear trend in LF ROM with types of avatar |
|  |  | β=-0.0508, effect size βz=-0.0449; pMC=0.3670 **=>** No sig. direct effect of types of avatar on ROM |
|  | Spinal functional ROM: Extension | β=0.0531, effect size βz=0.0473; pSM=0.1039, pPB=0.1116 **=>** No sig. relevant effects of types of avatar on extension |
|  |  | β=-0.1063, effect size βz=-0.0947; pSM=0.0115, pPB=0.0141 **=>** Small sig. negative effect of AOT on extension |
|  | Spinal functional ROM: HR | β=-0.0203, effect size βz=-0.0181; pSM=0.4870, pPB=0.5206 **=>** No sig. relevant effects of types of avatar on RH |
|  |  | β=0.0917, effect size βz=0.0817; pSM=0.0064, pPB=0.011 **=>** Small sig. positive effect of AOT on RH |
|  | **Virtual experience:** (LME analysis) | |
|  | Autonomous avatar Question: positive avatar characteristics (AAQ1) | β=0.0494, effect size βz=0.0440; pSM=0.2712, pPB=0.3030 **=>** No sig. effect of avatar and AOT to AAQ1 |
|  |  | effect size βz=0.1563; pSM=0.0082, pPB=0.0210 **=>** Small to medium effect of AAQ1 on LF-ROM |
|  |  | β=0.6637, effect size βz=0.5864; pSM<2×10(-16), pPB=0.0010 **=>** AAQ1 strongly dependent on types of avatar |

**Table 3b.** Cont.

| Barhorst-Cates et al. (2022) | **Virtual experience** | |
| --- | --- | --- |
|  | EQ | M=1.37 out of 2, SD=0.87 **=>** EG&CG have moderate level of embodiment |
|  |  | ps>0.3 **=>** No sig. effect of different points of view or populations on the level of embodiment |
|  |  | Highest score in EQ **=>** Better ownership of avatar in the first-person condition in EG&CG |
|  | **Other** | |
|  | Relationship between VR imitation accuracy and limb apraxia measure | χ2(2)=0.70, p=0.402  **=**> No sig. effect.  **=**> VR vs standard measures: VR is not sensitive to detect apraxia |
|  | Mental rotation accuracy | χ2(1)=6.23, p=0.013 **=>** Sig. effects of mental rotation task accuracy on IA |
|  |  | χ2(2)=6.32, p=0.043 **=>** Sig. effects of POV on imitation accuracy |
|  |  | B=0.3 **=>** Positive linear trend: Mental rotation accuracy↑, IA↑ |

**Table 3b.** Cont.

| Barhorst-Cates et | Mental rotation RT | χ2(1)=6.23, p=0.013 **=>** Sig. effects of mental rotation RT on IA |
| --- | --- | --- |
| al. (2022) |  | χ2(2)=12.49, p=0.002 **=>** Sig. effects of POV on IA |
|  |  | χ2(1)=0.10, p=0.748 **=>** No sig. effects of block-mirroring accuracy on IA |
|  |  | χ2(2)=8.96, p=0.011 **=>** Sig. effects of POV on IA |
|  | Block-mirroring RT | χ2(1)=0.27, p=0.606 **=>** No sig. effects of block-mirroring RT on IA |
|  |  | χ2(2)=8.95, p=0.011 **=>** Sig. effects of POV on IA |
|  | Block-matching accuracy | χ2(1)=0.01, p=0.916 **=>** No sig. effects of block-matching accuracy on IA |
|  |  | χ2(2)=8.95, p=0.011 **=>** Sig. effects of POV on IA |
|  | Block-matching RT | χ2(1)=12.07, p<0.001 **=>** Sig. effects of block-matching RT on IA |
|  |  | χ2(2)=8.93, p=0.011 **=>** Sig. effects of block-matching RT on IA |

**Table 3b.** Cont.

| Zhu, Y.et al. (2022) | **Functional** | |
| --- | --- | --- |
|  | Gait parameter: Stride | EG: 0.76±0.14; CG: 0.98±0.16 (p=0.003) **=>** CG>EG  **=>** sig. difference between CG & EG |
|  | Gait parameter: Step length | EG: 0.37±0.07; CG: 0.48±0.09 (p=0.01) **=>** CG>EG  **=>** sig. difference between CG & EG |
|  | Gait parameter: Step width | EG: 0.11±0.02; CG: 0.07±0.01 (p<0.001) **=>** CG>EG  **=>** sig. difference between CG & EG |
|  | Toe-out angle | EG: 7.64±1.63; CG: 6.43±0.67 (p<0.001) **=>** CG>EG  **=>** sig. difference between CG & EG |
|  | Gait speed | EG: 0.60±0.09; CG: 0.78±0.16 (p=0.006) **=>** CG>EG  **=>** sig. difference between CG & EG |
|  | Stance stage | EG: 64.35±3.72; CG: 60.01±1.15 (p=0.004) **=>** CG>EG  **=>** sig. difference between CG & EG |

| Sansoni et al. (2024) | **Psychological** | |
| --- | --- | --- |
|  | Subscales of EDI:  BU (pre-post) | CG: p>0.05 **=>** No sig. ↓ BU |
|  |  | EG: F(2,44)=3.27, p=0.047, η2p=0.13 **=>** Sig. ↓ episodes of binge eating and purging over time |
|  |  | EG: amd=-5.58, SE=1.7, p=0.006 **=>** Sig. ↓ BU |
|  | Subscales of EDI:  BU (1-month follow-up) | EG: amd=-6.5, SE=1.7, p=0.001 **=>** Sig. ↓ BU after 1 month |
|  | Subscales of EDI:  DT (pre-post) | CG: p>0.05 **=>** No sig. ↓ DT |
|  |  | EG: p>0.05 **=>** No sig. ↓ DT |
|  | Subscales of EDI:  DT (1-month follow-up) | EG: amd=-4.33, SE=1.23, p=0.003 **=>** Sig. ↓ preoccupation with weight and fear of weight gain after 1 month |
|  | Subscales of EDI:  BD (pre-post) | CG: p>0.05 **=>** No sig. ↓ BD |
|  |  | EG: p>0.05 **=>** No sig. ↓ BD |
|  |  | EG: F(1,21)=6.5, p=0.019, η2p=0.24 **=>** Sig. effect of baseline BMI on BD |

**Table 3b.** Cont.

| Sansoni et | **Physical** | |
| --- | --- | --- |
| al. (2024) | BMI (pre-post) | CG: amd=-1.58, SE=0.52, p=0.047 **=>** Sig. ↓ BMI |
|  |  | EG: amd=-1.59, SE=0.52, p=0.046 **=>** Sig. ↓ BMI |
|  | BMI (3-month follow-up vs 9-month follow-up) | CG: amd=-2.12, SE=0.52, p=0.001 **=>** Sig. ↓ BMI after 9 months vs 3 months |
|  | BMI (12-month follow-up) | CG: amd=-1.58, SE=0.52, p=0.047 **=>** Sig. ↓ BMI 12 months vs pre-test  **=>** (relapse BMI pattern during follow-up) |
|  |  | EG: amd=-1.59, SE=0.52, p=0.046 **=>** Sig. ↓ BMI 12 months vs pre-test  **=>** (stable BMI changing pattern during follow-up) |
| Xu et al. (2024) | **System embedded parameters:** Compare to CG1: No difference between CG1 & CG2 | |
|  | Group-Level ERD | Group-Level ERSP maps **=>** ERD within α & β band: E-MI>C-MI;  **=>** ERD (E-MI): α>β |
|  | IP HEMI:  peak ERD amplitude within α band | mean difference 8.69%, t(12)=3.076, p=0.01, d=0.829 **=>** E-MI>C-MI |
|  | IP HEMI:  peak ERD amplitude within β band | mean difference 4.33%, t(12)=2.273, p=0.042, d=0.630 **=>** E-MI>C-MI |

**Table 3b.** Cont.

| Xu et al. (2024) | CONTRA HEMI:  peak ERD amplitude within α band | mean difference 12.21%, t(12)=3.952, p=0.002, d=1.096 **=>** E-MI>C-MI |
| --- | --- | --- |
|  | CONTRA HEMI:  peak ERD amplitude within β band | mean difference 6.99%, t(12)=4.167, p=0.001, d=1.156 **=>** E-MI>C-MI |
| Mitchell et al. (2024) | **Functional** | |
|  | Physical activity | CG: Baseline: 105.5 (40.1);  CG: 6-month: 106.3 (45.4); p=0.02 **=>** Sig. ↓ |
|  |  | EG: Baseline: 106.4 (37.1);  EG: 6-month: 105.1 (46.9); p=0.02 **=>** Sig. ↓ |
|  |  | 3.1 (97.5% CI -6.9 to ∞ P<0.001) **=>** No sig. difference between EG & CG |
|  | **Physical** | |
|  | HbA1c (%) | CG: Baseline: 10.2 (1.8);  CG: 6-month: 9.4 (2.2); p<0.001 **=>** Sig.↑ |
|  |  | EG: Baseline: 9.7 (1.7);  EG: 6-month: 9.2 (2.1); p<0.001 **=>** Sig.↑ |
|  |  | 0.2 (97.5% CI -∞ to 0.3; P<0.001) **=>** No sig. difference between EG & CG |

| Mitchell | **Psychological** | |
| --- | --- | --- |
| et al. (2024) | Diabetes Distress Scale-17 | CG: pre vs 9-week: -0.4 (0.1); p<0.05;  pre vs 6-month: -0.2 (0.1) **=>** Sig. ↑ |
|  |  | CG: pre vs 9-week: -0.2 (10.1); p<0.05;  pre vs 6-month: -0.2 (0.1) **=>** Sig. ↑ |
|  |  | p=0.35; p=0.69 **=>** No sig. difference between EG & CG |

3PP: The third-person perspective; 8MM: Eight maker model; AOT: The amount of training; APAs: Anticipatory postural adjustments; Asyn: Asynchronous condition; AN: Anorexia nervosa; AOU: Amount of Use; BC: Body circumference; BD: Body Dissatisfaction; BMI: Body Mass Index; BI: Body image; BN: Bulimia nervosa; BPD: Borderline personality disorder; BW: Body width; BU: Bulimia; C-MI: Control motor imagery; CAVE: Cave automatic virtual environment; CBT: Cognitive behavioral therapy; CG: Control group; CP: Cerebral palsy; CONTRA: Contralesional; DOF: Degrees of freedom; DT: Drive for Thinness; EDI-2: Subscales of Eating Disorder Inventory-2; EG: Experimental group; ERD: Event-related desynchronization; ERSP: Event-related spectral perturbation map; E-MI: Enhanced motor imagery task; ES: Effect size; EQ: Embodiment Questionnaire; FBI: Full-body illusion; FMA: Fugl-Meyer Assessment; HbA1c: Hemoglobin A1c; HC: Healthy control(s); HD: Hemiplegia's disease; HEMI: Hemisphere; HBM: Human body model; HMD: Head-mounted display; IA: Imitation accuracy; IP: Ipsilesional; L: Left side; LF: Lateral flexion; LME: Linear mixed effects; LSSQ: Likert-scale specific questionnaire; MAL: Motor Activity Log; MAS: Modified Ashworth Scale; MBT: Mentalization-based group treatment; MI: Motor imagery; NHPT: Nine Hole Peg Test; No sig.: No significant; NRS: Numeric rating scale; PD: Parkinson’s disease; PDQ-39: Parkinson’s Disease Questionnaire, POV: Point of view; pPB = p-value with Parametric Bootstrapping; pSM: p-value with Saterthwaite Method; QoL: Quality of Life; R: Right side; ROM: Range of motion; RT: Reaction time; SES: Self-Esteem in Second Life; Significant: sig.; SOO: Sense of ownership; Syn: Synchronous condition; SF-36: 36-Item Short Form Health Survey; UPDRS: Unified Parkinson’s Disease Rating Scale; VR: Virtual reality; VRRS: Virtual reality rehabilitation system; HR: Horizontal rotation.
